# Transcriptional regulatory network refinement and quantification through kinetic modeling, gene expression microarray data and information theory

**DOI:** 10.1186/1471-2105-8-20

**Published:** 2007-01-23

**Authors:** Abdallah Sayyed-Ahmad, Kagan Tuncay, Peter J Ortoleva

**Affiliations:** 1 Center for Cell and Virus Theory, Department of Chemistry, Indiana University, Bloomington IN 47405, USA; 2 Department of Chemical Engineering and Materials Science, University of Minnesota, 421 Washington Ave SE, Minneapolis, MN 55455, USA

## Abstract

**Background:**

Gene expression microarray and other multiplex data hold promise for addressing the challenges of cellular complexity, refined diagnoses and the discovery of well-targeted treatments. A new approach to the construction and quantification of transcriptional regulatory networks (TRNs) is presented that integrates gene expression microarray data and cell modeling through information theory. Given a partial TRN and time series data, a probability density is constructed that is a functional of the time course of transcription factor (TF) thermodynamic activities at the site of gene control, and is a function of mRNA degradation and transcription rate coefficients, and equilibrium constants for TF/gene binding.

**Results:**

Our approach yields more physicochemical information that compliments the results of network structure delineation methods, and thereby can serve as an element of a comprehensive TRN discovery/quantification system. The most probable TF time courses and values of the aforementioned parameters are obtained by maximizing the probability obtained through entropy maximization. Observed time delays between mRNA expression and activity are accounted for implicitly since the time course of the activity of a TF is coupled by probability functional maximization, and is not assumed to be proportional to expression level of the mRNA type that translates into the TF. This allows one to investigate post-translational and TF activation mechanisms of gene regulation. Accuracy and robustness of the method are evaluated. A kinetic formulation is used to facilitate the analysis of phenomena with a strongly dynamical character while a physically-motivated regularization of the TF time course is found to overcome difficulties due to omnipresent noise and data sparsity that plague other methods of gene expression data analysis. An application to *Escherichia coli *is presented.

**Conclusion:**

Multiplex time series data can be used for the construction of the network of cellular processes and the calibration of the associated physicochemical parameters. We have demonstrated these concepts in the context of gene regulation understood through the analysis of gene expression microarray time series data. Casting the approach in a probabilistic framework has allowed us to address the uncertainties in gene expression microarray data. Our approach was found to be robust to error in the gene expression microarray data and mistakes in a proposed TRN.

## Background

Gene expression microarray [1-3] and other multiplex data (e.g. NMR, ChIP-on-chip and proteomics) contain a wealth of information, and thereby hold promise for addressing the challenge of cellular complexity and deriving advances in medical sciences [4-7]. Considering the volume of the data and the complexity of the phenomena to be understood, it is evident that the interpretation of such multiplex data must be facilitated by automation. Recently we proposed an approach to the analysis of multiplex bioanalytical data based on the integration of these data with cell modeling through information theory [8]. Here we show how this approach can be extended to the analysis of gene expression microarray time series data.

Kinetic cell models have been used for predicting cell behavior [9-11]. Unfortunately there is a lack of information about many of the rate and equilibrium constants for the reaction and transport processes involved [8,12]. Furthermore, we are presented with the challenge of calibrating and using an incomplete model since key aspects of biochemical networks have yet to be resolved. In contrast, gene expression microarray, protein spectroscopy, NMR, ChIP-on-chip and other multiplex data acquisition techniques yield many simultaneous measurements but they are often only indirectly related to the quantities we seek such as protein and mRNA production and degradation rate coefficients, and TF/gene binding constants, and the stoichiometry of posttranslational processes.

Time series experiments commonly involve monitoring a sample of cells over their cycle or during response to time-varying conditions in the extra-cellular medium such as due to heat shock, transitions to aerobic to anaerobic conditions, from enriched to minimal growth media, or exposure to hormones or drugs. Other dynamical phenomena of interest involve behaviors in response to nuclear transplantation, fertilization or viral infection, as well as the time course of normal development, radiation, transitions to abnormality or drug resistance. Predicting these phenomena, and analyzing of time series data on them can be facilitated using kinetic approaches if the associated dynamic variability is to be explored. In contrast, steady-state approaches can only yield ratios of rate coefficients and not all coefficients independently. Nor can a steady-state approach capture autonomous oscillatory dynamics such as observed during transcription [13,14].

To quantitatively understand the cell, we must account for the omnipresent uncertainty in observed data and in the structure of a cell model. Thus, a probabilistic framework is needed. We suggest that the probability of interest is a function of the rate parameters and initial concentrations and a functional of the time course of the frontier variables for which we do not know the governing equations or experimental measurements. Since we know the time course of gene expression microarray data, in principle, some of the rate parameters, equilibrium constants, initial concentrations as well as the time profile of the frontier variables are more likely to be consistent with it than others. A new approach to the construction and quantification of TRNs is presented here that integrates gene expression microarray time series data and cell modeling through information theory. Given a partial TRN and time series data, a probability density is constructed that is a functional of the time course of TF thermodynamic activities at the site of gene control, and is a function of mRNA degradation and transcription rate coefficients, and equilibrium constants for TF/gene binding.

In attempt to reduce the effect of measurement errors, gene expression microarray data is usually preprocessed via image analysis, statistical approaches and channel normalization before any biochemically viable information is derived [15-17]. A number of methods have been proposed for extracting information and overcoming systematic errors from gene expression microarray data after preprocessing it. Among them are Boolean network models [18], Bayesian network models [19], Bayesian statistics [20,21], cluster analysis [22,23], independent component analysis (ICA) [24,25], principal component analysis (PCA) [26,27] and network component analysis (NCA) [28,29]. These techniques are based on the assumption that the system is at steady-state.

The goal of Boolean model analysis is to infer gene regulatory network structure. However, Boolean network models oversimplify gene expression by using a binary approximation wherein genes are considered either active or inactive. The interaction between genes is then represented by Boolean functions (e.g. AND, OR, etc.), and hence the state of a gene (active/inactive) is calculated using the state of the controlling genes. A regulatory network is then constructed by searching all possible Boolean functions until a network that best fits all the data is obtained. While such approaches miss the subtler variation in the degree of gene activity, their computational efficiency allows them to be applied to large networks.

In Bayesian networks, the expression level of every gene is specified by a random variable. Starting form an *a priori *gene regulatory network, gene expression data and using Bayesian statistics, one can construct the conditional probability of the level of expression for each gene given the expression level of another gene that is assumed to regulate it. This conditional probability is then used to build a Bayesian network by keeping all edges (i.e. assumed regulatory interactions) that have a conditional probability higher than a threshold.

Cluster analysis, Bayesian statistics, ICA and PCA classify genes into groups; genes that have similar expression profiles are assumed to be similarly regulated or share the same biochemical functionality. However, they cannot uniquely predict the TRN as they do not address the role of TFs mediating gene-gene interactions or the effect of external factors (e.g. carbon source or TF activators/deactivators such as hormones). Cluster analysis is based on statistical techniques wherein correlations are sought between the responses of genes. However, the coordination can be extremely complex and circuitous. Thus genes may be part of a multi-branch feedback loop involving several TFs made or activated/deactivated by proteins translated from other genes via a series of kinetic steps that can introduce time delays which can easily mask some interactions or introduce spurious ones. Such effects are even more pronounced in light of noise in the observed expression profiles. Furthermore, for a given gene, there is no established correlation between mRNA expression and the level of protein it translates [30]. These time-delayed, complex relationships are revealed by our method which explicitly accounts for the role and time course of the TFs.

NCA differs from other techniques in that the structure of the TRN is assumed to be known. A number of assumptions are made in NCA to arrive at the final steady-state model. The approach presented here requires at least a part of the TRN. However, we place no restrictions on the structure of this network, use a kinetic model, construct synthetic gene expression microarray time series, apply a physically motivated regularization constraint for the time-dependence of TF activities that enhances robustness, places the entire computation in an information theory context so that the uncertainty can be assessed, and then analyzes TRN structure and the associated physicochemical parameters. The latter include mRNA production and degradation rates and TF/gene binding constants. The use of a kinetic model also allows us to generalize our approach to proteomic and metabolic data either by themselves or with gene expression microarray data.

Our method is significantly different from the approach proposed by Gardener *et al. *[31] whose objective is to construct the gene control network using gene expression microarray data and limiting the number of interactions per gene. However, even when there are just a few interactions per gene, there can be thousands of networks that can explain the same gene expression microarray data with a given accuracy. A variety of other methods has been also proposed for TRN construction and augmentation; these include gene ontology, phylogenetic profiles [32] and promoter sequence analysis [33]. The methodology presented here is meant to compliment other approaches and act as a filter for spurious networks by contrasting predictions with observed expression and predicting TF time courses. The later provides an important framework for TF/gene interactions or a self-consistency check on predictions of the other methods. Furthermore, as other methods suggest that there could be TF/gene interaction, our methodology compliments this by providing the specific nature (up/down) of the regulation.

In this paper, we present our approach and apply it to *E. coli*. Our analysis is based on a simplified approach wherein only TRN structure is obtained. Next the resulting TRN is used with the kinetic/information theory approach presented here to calibrate the physicochemical parameters and refine the network structure.

## Methods

Schematically, our approach to the incomplete-model challenge is as follows. The state of a cell is specified by a set of variables Ψ for which we know the governing equations and a set *T*, which is at the frontier of our understanding (i.e. for which we do not know the governing equations). The challenge is that the dynamics of Ψ is given by a cell model, e.g.

dΨ¯dt=G¯(Ψ¯,T¯,Λ¯),     (1)
 MathType@MTEF@5@5@+=feaafiart1ev1aaatCvAUfKttLearuWrP9MDH5MBPbIqV92AaeXatLxBI9gBaebbnrfifHhDYfgasaacH8akY=wiFfYdH8Gipec8Eeeu0xXdbba9frFj0=OqFfea0dXdd9vqai=hGuQ8kuc9pgc9s8qqaq=dirpe0xb9q8qiLsFr0=vr0=vr0dc8meaabaqaciaacaGaaeqabaqabeGadaaakeaadaWcaaqaaiabdsgaKjqbfI6azzaaDaaabaGaemizaqMaemiDaqhaaiabg2da9iqbdEeahzaaDaGaeiikaGIafuiQdKLba0bacqGGSaalcuWGubavgaqhaiabcYcaSiqbfU5amzaaDaGaeiykaKIaeiilaWIaaCzcaiaaxMaadaqadaqaaiabigdaXaGaayjkaiaawMcaaaaa@4173@

in which the rate *G *depends not only on many rate and equilibrium constants Λ, but also on the time-dependent frontier variables *T*(*t*). The descriptive variables, Ψ, can only be determined as a functional of the unknown time courses *T*(*t*). Thus the model cannot be simulated.

Gene expression microarray time series data, *M*, reflects the evolving state of the genome and hence one can compare it with a model predicted synthetic time series, *M*^*syn*^, to derive a measure of the accuracy and completeness of the model. Solving Eq. 1, yields the time-course of Ψ over the duration of the experiment as a function of Λ, the initial state Ψ(*t *= 0), and as a functional of *T*(*t*) (i.e. Ψ = Ψ[*T*,Λ,Ψ_0_]); thus the gene expression microarray data constructed, *M*^*syn*^, when compared with *M*, yields a measure of the accuracy of *T*(*t*), Λ and Ψ_0_. In the present application, Ψ represents intracellular mRNA levels, Λ is the aforementioned set of rate and binding constants, and *T*(*t*) is the time course of TF activities at the site of gene control.

To quantitatively understand the cell, we must account for the omnipresent uncertainty in observed data and in the structure of a cell model. Thus, a probabilistic framework is needed. We suggest that the probability of interest (denoted *ρ*) is a function of Λ and Ψ_0 _and a functional of the time course of the frontier variables *T*(*t*). Since we know *M*, in principle, some Λ, *T*(*t*) and Ψ_0 _are more likely to be consistent with it than others. We develop a model (e.g. a realization of Eq. 1) and use time series gene expression microarray data with information theory to construct *ρ*.

In the present approach the physics and chemistry of the mechanisms built into a kinetic cell model puts constraints on the relationship between Ψ and *T*(*t*), Λ and Ψ_0_; this facilitates the determination of these variables from time series data. In a sense, physics and chemistry of biochemical reaction/transport processes enhance the solvability of the inverse problem for the determination of *T*(*t*),Λ and Ψ_0 _given the time series gene expression microarray data. Since we know *M*, in principle, some Λ,*T*(*t*) and Ψ_0 _are more likely to be consistent with it than others. We develop a model (e.g. a realization of Eq. 1) and use time series gene expression microarray data with information theory to construct *ρ*.

### A transcription model

Gene expression is a multi-step process that is mainly regulated by proteins that activate or repress transcription. In prokaryotic genes, transcription initiation is controlled by promoters which are DNA sequence elements recognized by RNA polymerase. The activity of RNA polymerase is regulated through the interaction of the DNA-binding proteins known as transcription factors with short, specific DNA sequences. These sequences are normally located close to the promoter of the regulated gene. DNA-binding proteins alter the binding affinity of RNA polymerase, consequently affecting the RNA transcription rate [34]. It is evident that the physiological function of a DNA-binding protein is driven by its binding affinity with a gene promoter or adjacent DNA sequence. In particular, Repressor proteins bind to the promoter site thus competing with RNA polymerase for the same binding site while activator proteins usually bind adjacent to the promoter site and hence enhancing the binding affinity of RNA polymerase.

In our approach, we no longer require a comprehensive cell model to make quantitative predictions even though processes within and among the genome, proteome and metabolome are all strongly coupled. Rather our approach only requires an incomplete model wherein governing equations for the variables Ψ of Eq. 1 are to be set forth and the model must contain those variables with which one may construct synthetic data of the type available. Thus the following model is based on equations for mRNA levels so that synthetic gene expression microarray data can be constructed. But to do so one must know the time course of TFs as they up/down regulate genes. Specifically, to implement our approach the TF intra-nuclear thermodynamic activities are identified as the frontier variables *T*(*t*) of (Eq. 1). Closure is obtained by using time series data to generate functional differential equations for the *T*(*t*). Thus one need not make oversimplified assumptions on *T*(*t*) to compute Ψ (i.e. one may calibrate and run an incomplete transcription model even though the dynamical variables (e.g. RNA populations) are strongly coupled to *T*(*t*)).

We first develop a forward model in which given a set of time courses of TF thermodynamic activities, *T*(*t*), the time course of intra-cellular mRNA populations *R*(*t*) is predicted. Due to the dense environment within the nucleus, TF thermodynamic activities are preferred over concentrations. With such a model and time series gene expression microarray data, we now show how transcription rate and other parameters, the most probable *T*(*t*) can be obtained, and the gene control network can be quantitatively characterized. Finally, note that the following model is rather simple. While more complete models could be used [11], our purpose here is to demonstrate the approach and not to address the supercomputer challenge of a very detailed approach.

Given a TRN with *N*_*TF *_TFs and *N*_*g *_genes, the *i*-th gene (g_i_), *i *= 1,⋯*N*_*g*_, is assumed to have Ns(i)
 MathType@MTEF@5@5@+=feaafiart1ev1aaatCvAUfKttLearuWrP9MDH5MBPbIqV92AaeXatLxBI9gBaebbnrfifHhDYfgasaacH8akY=wiFfYdH8Gipec8Eeeu0xXdbba9frFj0=OqFfea0dXdd9vqai=hGuQ8kuc9pgc9s8qqaq=dirpe0xb9q8qiLsFr0=vr0=vr0dc8meaabaqaciaacaGaaeqabaqabeGadaaakeaacqWGobGtdaqhaaWcbaGaem4CamhabaGaeiikaGIaemyAaKMaeiykaKcaaaaa@327A@ uncompetitive TF binding sites labeled *j *= 1,⋯Ns(i)
 MathType@MTEF@5@5@+=feaafiart1ev1aaatCvAUfKttLearuWrP9MDH5MBPbIqV92AaeXatLxBI9gBaebbnrfifHhDYfgasaacH8akY=wiFfYdH8Gipec8Eeeu0xXdbba9frFj0=OqFfea0dXdd9vqai=hGuQ8kuc9pgc9s8qqaq=dirpe0xb9q8qiLsFr0=vr0=vr0dc8meaabaqaciaacaGaaeqabaqabeGadaaakeaacqWGobGtdaqhaaWcbaGaem4CamhabaGaeiikaGIaemyAaKMaeiykaKcaaaaa@327A@. Assume the binding at any site is independent of the state of others and that only one type of TFs can bind to each site. While the latter two assumptions are not inherent restrictions of our approach, they are made here for simplicity. Let *n*_*ij *_label the TF that can bind to site *j *on gene *i*. Let *b*_*ij *_be minus one or plus one indicate the nature of regulation (down or up) of TF type *n*_*ij *_on g_i_. At TF/gene equilibrium, the probability *H*_*i *_that g_i _is available for RNA polymerase (RP) complexing is taken to be given by an equilibrium Langmuir uncompetitive adsorption isotherm [35]

Hi=∏j=1Ns(i)(QijTnij)(1+bij2)/(1+QijTnij),     (2)
 MathType@MTEF@5@5@+=feaafiart1ev1aaatCvAUfKttLearuWrP9MDH5MBPbIqV92AaeXatLxBI9gBaebbnrfifHhDYfgasaacH8akY=wiFfYdH8Gipec8Eeeu0xXdbba9frFj0=OqFfea0dXdd9vqai=hGuQ8kuc9pgc9s8qqaq=dirpe0xb9q8qiLsFr0=vr0=vr0dc8meaabaqaciaacaGaaeqabaqabeGadaaakeaacqWGibasdaWgaaWcbaGaemyAaKgabeaakiabg2da9maalyaabaWaaebCaeaadaqadaqaaiabdgfarnaaBaaaleaacqWGPbqAcqWGQbGAaeqaaOGaemivaq1aaSbaaSqaaiabd6gaUnaaBaaameaacqWGPbqAcqWGQbGAaeqaaaWcbeaaaOGaayjkaiaawMcaamaaCaaaleqabaWaaeWaaeaadaWcaaqaaiabigdaXiabgUcaRiabdkgaInaaBaaameaacqWGPbqAcqWGQbGAaeqaaaWcbaGaeGOmaidaaaGaayjkaiaawMcaaaaaaeaacqWGQbGAcqGH9aqpcqaIXaqmaeaacqWGobGtdaqhaaadbaGaem4CamhabaGaeiikaGIaemyAaKMaeiykaKcaaaqdcqGHpis1aaGcbaWaaeWaaeaacqaIXaqmcqGHRaWkcqWGrbqudaWgaaWcbaGaemyAaKMaemOAaOgabeaakiabdsfaunaaBaaaleaacqWGUbGBdaWgaaadbaGaemyAaKMaemOAaOgabeaaaSqabaaakiaawIcacaGLPaaaaaGaeiilaWIaaCzcaiaaxMaadaqadaqaaiabikdaYaGaayjkaiaawMcaaaaa@61B8@

for binding constant *Q*_*ij *_(liter/mole) and intra-nuclear activity Tnij
 MathType@MTEF@5@5@+=feaafiart1ev1aaatCvAUfKttLearuWrP9MDH5MBPbIqV92AaeXatLxBI9gBaebbnrfifHhDYfgasaacH8akY=wiFfYdH8Gipec8Eeeu0xXdbba9frFj0=OqFfea0dXdd9vqai=hGuQ8kuc9pgc9s8qqaq=dirpe0xb9q8qiLsFr0=vr0=vr0dc8meaabaqaciaacaGaaeqabaqabeGadaaakeaacqWGubavdaWgaaWcbaGaemOBa42aaSbaaWqaaiabdMgaPjabdQgaQbqabaaaleqaaaaa@325E@ of the *n*_*ij*_-th TF. The rate of RNA transcription initiation is written

ki=kimax⁡Hi(Q¯¯,b¯¯,T¯),     (3)
 MathType@MTEF@5@5@+=feaafiart1ev1aaatCvAUfKttLearuWrP9MDH5MBPbIqV92AaeXatLxBI9gBaebbnrfifHhDYfgasaacH8akY=wiFfYdH8Gipec8Eeeu0xXdbba9frFj0=OqFfea0dXdd9vqai=hGuQ8kuc9pgc9s8qqaq=dirpe0xb9q8qiLsFr0=vr0=vr0dc8meaabaqaciaacaGaaeqabaqabeGadaaakeaacqWGRbWAdaWgaaWcbaGaemyAaKgabeaakiabg2da9iabdUgaRnaaDaaaleaacqWGPbqAaeaacyGGTbqBcqGGHbqycqGG4baEaaGccqWGibasdaWgaaWcbaGaemyAaKgabeaakmaabmaabaGafmyuaeLba0Hba0bacqGGSaalcuWGIbGygaqhgaqhaiabcYcaSiqbdsfauzaaDaaacaGLOaGaayzkaaGaeiilaWIaaCzcaiaaxMaadaqadaqaaiabiodaZaGaayjkaiaawMcaaaaa@46A8@

where kimax⁡
 MathType@MTEF@5@5@+=feaafiart1ev1aaatCvAUfKttLearuWrP9MDH5MBPbIqV92AaeXatLxBI9gBaebbnrfifHhDYfgasaacH8akY=wiFfYdH8Gipec8Eeeu0xXdbba9frFj0=OqFfea0dXdd9vqai=hGuQ8kuc9pgc9s8qqaq=dirpe0xb9q8qiLsFr0=vr0=vr0dc8meaabaqaciaacaGaaeqabaqabeGadaaakeaacqWGRbWAdaqhaaWcbaGaemyAaKgabaGagiyBa0MaeiyyaeMaeiiEaGhaaaaa@33B9@ is a saturation rate coefficient that we suggest is diffusion-limited. This assumption is reasonable because it take into account that on one hand, for TF/DNA and RP/DNA binding, electrostatic interactions tend to lead to higher limiting rate coefficient than the diffusion limited one. On the other hand, the need for having a specific orientation in order for a TF or RP to bind to the DNA tends to lower the limiting rate coefficient than the diffusion limited one [36]. The two aforementioned processes in addition to electrostatic screening due to presence of salt in *vivo *are assumed to balance each other. After RP binds to a gene mRNA elongation commences. If nucleotide concentrations are roughly steady during transcription and the RP advancement velocity *u*_*i *_(which in principle depends on the sequence of the gene and measured in units of nucleotides/sec), then the transcription polymerization rate *A*_*i *_is taken to be

1Ai=1ki[RP]+Nnuciui.     (4)
 MathType@MTEF@5@5@+=feaafiart1ev1aaatCvAUfKttLearuWrP9MDH5MBPbIqV92AaeXatLxBI9gBaebbnrfifHhDYfgasaacH8akY=wiFfYdH8Gipec8Eeeu0xXdbba9frFj0=OqFfea0dXdd9vqai=hGuQ8kuc9pgc9s8qqaq=dirpe0xb9q8qiLsFr0=vr0=vr0dc8meaabaqaciaacaGaaeqabaqabeGadaaakeaadaWcaaqaaiabigdaXaqaaiabdgeabnaaBaaaleaacqWGPbqAaeqaaaaakiabg2da9maalaaabaGaeGymaedabaGaem4AaS2aaSbaaSqaaiabdMgaPbqabaGccqGGBbWwcqWGsbGucqWGqbaucqGGDbqxaaGaey4kaSYaaSaaaeaacqWGobGtdaqhaaWcbaGaemOBa4MaemyDauNaem4yamgabaGaemyAaKgaaaGcbaGaemyDau3aaSbaaSqaaiabdMgaPbqabaaaaOGaeiOla4IaaCzcaiaaxMaadaqadaqaaiabisda0aGaayjkaiaawMcaaaaa@498F@

A form which captures the rate limiting step of the two serial processes (RP binding and the elongation). With this, the governing equation for the mRNA populations is written as

dRidt=Ai−λiRi,     (5)
 MathType@MTEF@5@5@+=feaafiart1ev1aaatCvAUfKttLearuWrP9MDH5MBPbIqV92AaeXatLxBI9gBaebbnrfifHhDYfgasaacH8akY=wiFfYdH8Gipec8Eeeu0xXdbba9frFj0=OqFfea0dXdd9vqai=hGuQ8kuc9pgc9s8qqaq=dirpe0xb9q8qiLsFr0=vr0=vr0dc8meaabaqaciaacaGaaeqabaqabeGadaaakeaadaWcaaqaaiabdsgaKjabdkfasnaaBaaaleaacqWGPbqAaeqaaaGcbaGaemizaqMaemiDaqhaaiabg2da9iabdgeabnaaBaaaleaacqWGPbqAaeqaaOGaeyOeI0ccciGae83UdW2aaSbaaSqaaiabdMgaPbqabaGccqWGsbGudaWgaaWcbaGaemyAaKgabeaakiabcYcaSiaaxMaacaWLjaWaaeWaaeaacqaI1aqnaiaawIcacaGLPaaaaaa@42CB@

where Nnuci
 MathType@MTEF@5@5@+=feaafiart1ev1aaatCvAUfKttLearuWrP9MDH5MBPbIqV92AaeXatLxBI9gBaebbnrfifHhDYfgasaacH8akY=wiFfYdH8Gipec8Eeeu0xXdbba9frFj0=OqFfea0dXdd9vqai=hGuQ8kuc9pgc9s8qqaq=dirpe0xb9q8qiLsFr0=vr0=vr0dc8meaabaqaciaacaGaaeqabaqabeGadaaakeaacqWGobGtdaqhaaWcbaGaemOBa4MaemyDauNaem4yamgabaGaemyAaKgaaaaa@3380@ is the total length of the gene g_*i*_, and *λ*_*i *_is the decay constant. However, for a more detailed model, mRNA degradation could depend on mRNA protein binding factors as well as the level of some hormones or metabolites such as iron [37]. If the rate limiting step for transcription is RNA polymerase binding to the gene [34], then the second term in Eq. 4 may be dropped. Finally, [*RP*] is the activity of free RNA polymerase and is assumed to be constant and henceforth is subsumed in kimax⁡
 MathType@MTEF@5@5@+=feaafiart1ev1aaatCvAUfKttLearuWrP9MDH5MBPbIqV92AaeXatLxBI9gBaebbnrfifHhDYfgasaacH8akY=wiFfYdH8Gipec8Eeeu0xXdbba9frFj0=OqFfea0dXdd9vqai=hGuQ8kuc9pgc9s8qqaq=dirpe0xb9q8qiLsFr0=vr0=vr0dc8meaabaqaciaacaGaaeqabaqabeGadaaakeaacqWGRbWAdaqhaaWcbaGaemyAaKgabaGagiyBa0MaeiyyaeMaeiiEaGhaaaaa@33B9@. The governing equation for mRNA levels evolution becomes

dRidt=kimax⁡Hi(Q¯¯,b¯¯,T¯)−λiRi.     (6)
 MathType@MTEF@5@5@+=feaafiart1ev1aaatCvAUfKttLearuWrP9MDH5MBPbIqV92AaeXatLxBI9gBaebbnrfifHhDYfgasaacH8akY=wiFfYdH8Gipec8Eeeu0xXdbba9frFj0=OqFfea0dXdd9vqai=hGuQ8kuc9pgc9s8qqaq=dirpe0xb9q8qiLsFr0=vr0=vr0dc8meaabaqaciaacaGaaeqabaqabeGadaaakeaadaWcaaqaaiabdsgaKjabdkfasnaaBaaaleaacqWGPbqAaeqaaaGcbaGaemizaqMaemiDaqhaaiabg2da9iabdUgaRnaaDaaaleaacqWGPbqAaeaacyGGTbqBcqGGHbqycqGG4baEaaGccqWGibasdaWgaaWcbaGaemyAaKgabeaakmaabmaabaGafmyuaeLba0Hba0bacqGGSaalcuWGIbGygaqhgaqhaiabcYcaSiqbdsfauzaaDaaacaGLOaGaayzkaaGaeyOeI0ccciGae83UdW2aaSbaaSqaaiabdMgaPbqabaGccqWGsbGudaWgaaWcbaGaemyAaKgabeaakiabc6caUiaaxMaacaWLjaWaaeWaaeaacqaI2aGnaiaawIcacaGLPaaaaaa@519A@

Finally, our methodology can be generalized by relaxing any of the above assumptions. For example, *H*_*i*_, can be changed such that competitive binding and TF complexing are accounted for explicitly which in effect will allow for OR logic. Although the extension of our transcription model to include competitive binding is crucial to accurately recover TF activity time courses, this level of description is out of the scope of this study. Further research is needed to obtain specific data on the molecular level about which TF binds to which binding site of a given gene.

### Information theory model/data integration

#### General formulation

Gene expression microarray data is fraught with inaccuracies. Much attention has been placed on minimizing systematic and random errors via quality screening, multi-spot/multi-slide analysis and averaging. Software carrying out these functions yields confidence intervals which are quantitative measures of errors in the experimental data. Information theory was introduced as a method for assessing the uncertainty in the state of a system via an entropy measure [38,39]. In a series of papers [8,40], we have shown how information theory can be used to calibrate model parameters, use an incomplete model and estimate the associated uncertainties based on the inaccuracies in the observed data and the model used. The probability density *ρ *for the values of the set Λ of model parameters and the time-dependence of *T*(*t*) (the set of variables whose governing equations are not in the model, here TF activities within the nucleus) is obtained through entropy maximization.

The development starts with the introduction of the entropy *S*,

S=−SΛ¯,T¯ρln⁡ρ,     (7)
 MathType@MTEF@5@5@+=feaafiart1ev1aaatCvAUfKttLearuWrP9MDH5MBPbIqV92AaeXatLxBI9gBamrtHrhAL1wy0L2yHvtyaeHbnfgDOvwBHrxAJfwnaebbnrfifHhDYfgasaacH8akY=wiFfYdH8Gipec8Eeeu0xXdbba9frFj0=OqFfea0dXdd9vqai=hGuQ8kuc9pgc9s8qqaq=dirpe0xb9q8qiLsFr0=vr0=vr0dc8meaabaqaciaacaGaaeqabaWaaeGaeaaakeaacqWGtbWucqGH9aqpcqGHsisldaWfqaqaaGWabiab=jr8tbWcbaGafu4MdWKba0bacqGGSaalcuWGubavgaqhaaqabaacciGccqGFbpGCcyGGSbaBcqGGUbGBcqGFbpGCcqGGSaalcaWLjaGaaCzcamaabmaabaGaeG4naCdacaGLOaGaayzkaaaaaa@4A68@

where S
 MathType@MTEF@5@5@+=feaafiart1ev1aaatCvAUfKttLearuWrP9MDH5MBPbIqV92AaeXatLxBI9gBamrtHrhAL1wy0L2yHvtyaeHbnfgDOvwBHrxAJfwnaebbnrfifHhDYfgasaacH8akY=wiFfYdH8Gipec8Eeeu0xXdbba9frFj0=OqFfea0dXdd9vqai=hGuQ8kuc9pgc9s8qqaq=dirpe0xb9q8qiLsFr0=vr0=vr0dc8meaabaqaciaacaGaaeqabaWaaeGaeaaakeaaimqacqWFse=uaaa@3846@ is an integration over all Λ and a functional integration over all time courses *T*(*t*) is indicated. The experimental data and model are introduced via a set of error measures labeled *l *= 1,2,..., each of whose average E¯l
 MathType@MTEF@5@5@+=feaafiart1ev1aaatCvAUfKttLearuWrP9MDH5MBPbIqV92AaeXatLxBI9gBaebbnrfifHhDYfgasaacH8akY=wiFfYdH8Gipec8Eeeu0xXdbba9frFj0=OqFfea0dXdd9vqai=hGuQ8kuc9pgc9s8qqaq=dirpe0xb9q8qiLsFr0=vr0=vr0dc8meaabaqaciaacaGaaeqabaqabeGadaaakeaacuWGfbqrgaqeamaaCaaaleqabaGaemiBaWgaaaaa@2F65@ is assumed known and are given in terms of *ρ *via

SΛ¯,T¯ρEl=E¯l.     (8)
 MathType@MTEF@5@5@+=feaafiart1ev1aaatCvAUfKttLearuWrP9MDH5MBPbIqV92AaeXatLxBI9gBamrtHrhAL1wy0L2yHvtyaeHbnfgDOvwBHrxAJfwnaebbnrfifHhDYfgasaacH8akY=wiFfYdH8Gipec8Eeeu0xXdbba9frFj0=OqFfea0dXdd9vqai=hGuQ8kuc9pgc9s8qqaq=dirpe0xb9q8qiLsFr0=vr0=vr0dc8meaabaqaciaacaGaaeqabaWaaeGaeaaakeaadaWfqaqaaGWabiab=jr8tbWcbaGafu4MdWKba0bacqGGSaalcuWGubavgaqhaaqabaacciGccqGFbpGCcqWGfbqrdaahaaWcbeqaaiabdYgaSbaakiabg2da9iqbdweafzaaraWaaWbaaSqabeaacqWGSbaBaaGccqGGUaGlcaWLjaGaaCzcamaabmaabaGaeGioaGdacaGLOaGaayzkaaaaaa@4940@

For *cDNA *microarray data, E¯cDNA
 MathType@MTEF@5@5@+=feaafiart1ev1aaatCvAUfKttLearuWrP9MDH5MBPbIqV92AaeXatLxBI9gBaebbnrfifHhDYfgasaacH8akY=wiFfYdH8Gipec8Eeeu0xXdbba9frFj0=OqFfea0dXdd9vqai=hGuQ8kuc9pgc9s8qqaq=dirpe0xb9q8qiLsFr0=vr0=vr0dc8meaabaqaciaacaGaaeqabaqabeGadaaakeaacuWGfbqrgaqeamaaCaaaleqabaGaem4yamMaemiraqKaemOta4Kaemyqaeeaaaaa@3294@ can be estimated from confidence intervals provided by statistical data analysis. According to the information theory prescription we construct *ρ*(*T*(*t*),Λ) by maximizing *S *subject to the constraints (Eq. 8), normalization of *ρ*, qualitative information on the timescale over which *T*(*t*) can evolve, and other factors reflecting one's expertise. The result is a form for *ρ *which implies that the Λ, *T*(*t*) which are most probable yield the lowest error. Also the *T*(*t*) obtained has no time dependence that is unphysically short. Other constraints could be introduced that allow one to assign higher probability to the range of parameter values Λ that are near those one expects from experience. Given the inherently subjective nature of probability (e.g. if we know nothing then all Λ, *T*(*t*) are equally likely), the information theory prescription yields a *ρ *that is to be consistent with the level of our knowledge of the system. In our procedure, the resulting *ρ *is then maximized with respect to Λ and *T*(*t*) to determine their most probable values. Cell parameters that must be calibrated to attain a predictive model using our approach are those introduced in the previous section.

### Implementation for cDNA microarray data

Our analysis starts with preprocessed data, thus the predictions of our method as other genome wide microarray analysis methods depend on the choice of the preprocessing procedure (although the approach could also be generalized to proceed directly with raw data). Analysis of preprocessed microarray data can be placed in our framework by introducing an associated error *E*^*cDNA*^. Let *M*_*i*ℓ _be the microarray expression level for the *i*-th of *N*_*g *_genes in the ℓ-th of *N*_*micro *_experiments (e.g. time slice). Then

EcDNA=∑ℓ=1Nmicro∑i=1Ng(ln⁡miℓsyn−ln⁡miℓobs)2,     (9)
 MathType@MTEF@5@5@+=feaafiart1ev1aaatCvAUfKttLearuWrP9MDH5MBPbIqV92AaeXatLxBI9gBaebbnrfifHhDYfgasaacH8akY=wiFfYdH8Gipec8Eeeu0xXdbba9frFj0=OqFfea0dXdd9vqai=hGuQ8kuc9pgc9s8qqaq=dirpe0xb9q8qiLsFr0=vr0=vr0dc8meaabaqaciaacaGaaeqabaqabeGadaaakeaacqWGfbqrdaahaaWcbeqaaiabdogaJjabdseaejabd6eaojabdgeabbaakiabg2da9maaqahabaWaaabCaeaadaqadaqaaiGbcYgaSjabc6gaUjabd2gaTnaaDaaaleaacqWGPbqAcqWItecBaeaacqWGZbWCcqWG5bqEcqWGUbGBaaGccqGHsislcyGGSbaBcqGGUbGBcqWGTbqBdaqhaaWcbaGaemyAaKMaeS4eHWgabaGaem4Ba8MaemOyaiMaem4CamhaaaGccaGLOaGaayzkaaWaaWbaaSqabeaacqaIYaGmaaaabaGaemyAaKMaeyypa0JaeGymaedabaGaemOta40aaSbaaWqaaiabdEgaNbqabaaaniabggHiLdaaleaacqWItecBcqGH9aqpcqaIXaqmaeaacqWGobGtdaWgaaadbaGaemyBa0MaemyAaKMaem4yamMaemOCaiNaem4Ba8gabeaaa0GaeyyeIuoakiabcYcaSiaaxMaacaWLjaWaaeWaaeaacqaI5aqoaiaawIcacaGLPaaaaaa@67EF@

where *m*_*i*ℓ _= *M*_*i*ℓ_/*M*_*iA *_with *A *being the initial time or the standard condition. Here, miℓsyn
 MathType@MTEF@5@5@+=feaafiart1ev1aaatCvAUfKttLearuWrP9MDH5MBPbIqV92AaeXatLxBI9gBaebbnrfifHhDYfgasaacH8akY=wiFfYdH8Gipec8Eeeu0xXdbba9frFj0=OqFfea0dXdd9vqai=hGuQ8kuc9pgc9s8qqaq=dirpe0xb9q8qiLsFr0=vr0=vr0dc8meaabaqaciaacaGaaeqabaqabeGadaaakeaacqWGTbqBdaqhaaWcbaGaemyAaKMaeS4eHWgabaGaem4CamNaemyEaKNaemOBa4gaaaaa@3517@ is the synthetic microarray data constructed from mRNA levels predicted from a cell model (e.g. here that of the previous section), while *obs *indicates an observed value. Thus *E*^*cDNA *^is a function of the set of model parameters Λ as contained in a cell model (e.g. Q¯¯
 MathType@MTEF@5@5@+=feaafiart1ev1aaatCvAUfKttLearuWrP9MDH5MBPbIqV92AaeXatLxBI9gBaebbnrfifHhDYfgasaacH8akY=wiFfYdH8Gipec8Eeeu0xXdbba9frFj0=OqFfea0dXdd9vqai=hGuQ8kuc9pgc9s8qqaq=dirpe0xb9q8qiLsFr0=vr0=vr0dc8meaabaqaciaacaGaaeqabaqabeGadaaakeaacuWGrbqugaqhgaqhaaaa@2E1E@,*k*,*λ* and b¯¯
 MathType@MTEF@5@5@+=feaafiart1ev1aaatCvAUfKttLearuWrP9MDH5MBPbIqV92AaeXatLxBI9gBaebbnrfifHhDYfgasaacH8akY=wiFfYdH8Gipec8Eeeu0xXdbba9frFj0=OqFfea0dXdd9vqai=hGuQ8kuc9pgc9s8qqaq=dirpe0xb9q8qiLsFr0=vr0=vr0dc8meaabaqaciaacaGaaeqabaqabeGadaaakeaacuWGIbGygaqhgaqhaaaa@2E40@). Similarly *E*^*cDNA *^is a functional of the time course *T*(*t*) of intra-nuclear TF activities. Following the above information theory formulation we introduce the probability *ρ *= *ρ *(*T*(*t*), Λ), a functional of the time course *T*(*t*) and a function of Λ. We construct *ρ *by maximizing the entropy subject to estimates of the average error measures (here *E*^*cDNA*^) and other information.

The number of time points is restricted due to cost. This fact and the high level of uncertainty in microarray data suggest that the probability functional method cannot yield a meaningful *T*(*t*) unless more information is known. In our formulation this is introduced via a homogenization constraint that eliminates unphysically short timescale variations in *T*(*t*) that the sparseness of the time series data would otherwise allow. In particular, we impose the constraint

SΛ¯,T¯ρ∫0tf(dTndt)2dt=tf/Q¯2tc2,     (10)
MathType@MTEF@5@5@+=feaafiart1ev1aaatCvAUfKttLearuWrP9MDH5MBPbIqV92AaeXatLxBI9gBamrtHrhAL1wy0L2yHvtyaeHbnfgDOvwBHrxAJfwnaebbnrfifHhDYfgasaacH8akY=wiFfYdH8Gipec8Eeeu0xXdbba9frFj0=OqFfea0dXdd9vqai=hGuQ8kuc9pgc9s8qqaq=dirpe0xb9q8qiLsFr0=vr0=vr0dc8meaabaqaciaacaGaaeqabaWaaeGaeaaakeaadaWfqaqaaGWabiab=jr8tbWcbaGafu4MdWKba0bacqGGSaalcuWGubavgaqhaaqabaacciGccqGFbpGCdaWdXbqaamaabmaabaWaaSaaaeaacqWGKbazcqWGubavdaWgaaWcbaGaemOBa4gabeaaaOqaaiabdsgaKjabdsha0baaaiaawIcacaGLPaaadaahaaWcbeqaaiabikdaYaaaaeaacqaIWaamaeaacqWG0baDdaWgaaadbaGaemOzaygabeaaa0Gaey4kIipakmaalyaabaGaemizaqMaemiDaqNaeyypa0JaemiDaq3aaSbaaSqaaiabdAgaMbqabaaakeaacuWGrbqugaqeamaaCaaaleqabaGaeGOmaidaaOGaemiDaq3aa0baaSqaaiabdogaJbqaaiabikdaYaaaaaGccqGGSaalcaWLjaGaaCzcamaabmaabaGaeGymaeJaeGimaadacaGLOaGaayzkaaaaaa@60B7@

for a time series run over the interval from 0 to *t*_*f*_;*t*_*c *_is the shortest characteristic time over which we expect that *T*(*t*) can change appreciably and we assume Q¯
 MathType@MTEF@5@5@+=feaafiart1ev1aaatCvAUfKttLearuWrP9MDH5MBPbIqV92AaeXatLxBI9gBaebbnrfifHhDYfgasaacH8akY=wiFfYdH8Gipec8Eeeu0xXdbba9frFj0=OqFfea0dXdd9vqai=hGuQ8kuc9pgc9s8qqaq=dirpe0xb9q8qiLsFr0=vr0=vr0dc8meaabaqaciaacaGaaeqabaqabeGadaaakeaacuWGrbqugaqeaaaa@2DEF@ (the average TF/gene binding constant) is the inverse of the typical variation of *T*.

One can also use a steady-state approximation for information available about post-translational reactions to further constrain *S*. If a subnetwork of genes with stoichiometric matrix of processes *d*_*n *_is responsible for the production of *T*_*n*_, then the associated error measure for these processes is

ETF=∑n=1NTF∑k=1Ntimes(Tn(tk)−αn∏j=1znmdnjobs(tk))2,     (11)
 MathType@MTEF@5@5@+=feaafiart1ev1aaatCvAUfKttLearuWrP9MDH5MBPbIqV92AaeXatLxBI9gBaebbnrfifHhDYfgasaacH8akY=wiFfYdH8Gipec8Eeeu0xXdbba9frFj0=OqFfea0dXdd9vqai=hGuQ8kuc9pgc9s8qqaq=dirpe0xb9q8qiLsFr0=vr0=vr0dc8meaabaqaciaacaGaaeqabaqabeGadaaakeaacqWGfbqrdaahaaWcbeqaaiabdsfaujabdAeagbaakiabg2da9maaqahabaWaaabCaeaadaqadaqaaiabdsfaunaaBaaaleaacqWGUbGBaeqaaOWaaeWaaeaacqWG0baDdaWgaaWcbaGaem4AaSgabeaaaOGaayjkaiaawMcaaiabgkHiTGGaciab=f7aHnaaBaaaleaacqWGUbGBaeqaaOWaaebCaeaacqWGTbqBdaqhaaWcbaGaemizaq2aaSbaaWqaaiabd6gaUjabdQgaQbqabaaaleaacqWGVbWBcqWGIbGycqWGZbWCaaGcdaqadaqaaiabdsha0naaBaaaleaacqWGRbWAaeqaaaGccaGLOaGaayzkaaaaleaacqWGQbGAcqGH9aqpcqaIXaqmaeaacqWG6bGEdaWgaaadbaGaemOBa4gabeaaa0Gaey4dIunaaOGaayjkaiaawMcaamaaCaaaleqabaGaeGOmaidaaaqaaiabdUgaRjabg2da9iabigdaXaqaaiabd6eaonaaBaaameaacqWG0baDcqWGPbqAcqWGTbqBcqWGLbqzcqWGZbWCaeqaaaqdcqGHris5aaWcbaGaemOBa4Maeyypa0JaeGymaedabaGaemOta40aaSbaaWqaaiabdsfaujabdAeagbqabaaaniabggHiLdGccqGGSaalcaWLjaGaaCzcamaabmaabaGaeGymaeJaeGymaedacaGLOaGaayzkaaaaaa@7342@

where *N*_*times *_is the number of discretized times at which the TF activity is computed, *z*_*n *_is the number of genes involved in the production of *T*_*n*_, and *α*_*n *_is an equilibrium constant. mdnjobs
 MathType@MTEF@5@5@+=feaafiart1ev1aaatCvAUfKttLearuWrP9MDH5MBPbIqV92AaeXatLxBI9gBaebbnrfifHhDYfgasaacH8akY=wiFfYdH8Gipec8Eeeu0xXdbba9frFj0=OqFfea0dXdd9vqai=hGuQ8kuc9pgc9s8qqaq=dirpe0xb9q8qiLsFr0=vr0=vr0dc8meaabaqaciaacaGaaeqabaqabeGadaaakeaacqWGTbqBdaqhaaWcbaGaemizaq2aaSbaaWqaaiabd6gaUjabdQgaQbqabaaaleaacqWGVbWBcqWGIbGycqWGZbWCaaaaaa@36AA@ is the observed microarray for the *j*-th gene responsible for the creation of the *n*-th TF.

**Figure 1:**
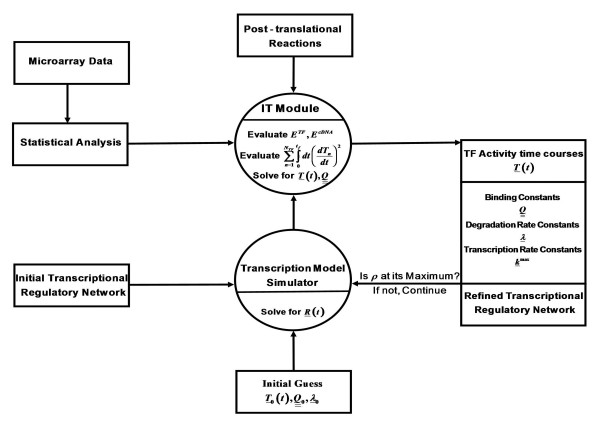
A flowchart of our transcription model/microarray data integration.

Maximization of the entropy with respect to *ρ *gives

ln⁡ρ=ln⁡Ξ−β1EcDNA−β2ETF−ω∑n=1NTF∫0tfdt(dTndt)2,     (12)
MathType@MTEF@5@5@+=feaafiart1ev1aaatCvAUfKttLearuWrP9MDH5MBPbIqV92AaeXatLxBI9gBaebbnrfifHhDYfgasaacH8akY=wiFfYdH8Gipec8Eeeu0xXdbba9frFj0=OqFfea0dXdd9vqai=hGuQ8kuc9pgc9s8qqaq=dirpe0xb9q8qiLsFr0=vr0=vr0dc8meaabaqaciaacaGaaeqabaqabeGadaaakeaacyGGSbaBcqGGUbGBiiGacqWFbpGCcqGH9aqpcyGGSbaBcqGGUbGBcqqHEoawcqGHsislcqWFYoGydaWgaaWcbaGaeGymaedabeaakiabdweafnaaCaaaleqabaGaem4yamMaemiraqKaemOta4KaemyqaeeaaOGaeyOeI0Iae8NSdi2aaSbaaSqaaiabikdaYaqabaGccqWGfbqrdaahaaWcbeqaaiabdsfaujabdAeagbaakiabgkHiTiab=L8a3naaqahabaWaa8qCaeaacqWGKbazcqWG0baDaSqaaiabicdaWaqaaiabdsha0naaBaaameaacqWGMbGzaeqaaaqdcqGHRiI8aaWcbaGaemOBa4Maeyypa0JaeGymaedabaGaemOta40aaSbaaWqaaiabdsfaujabdAeagbqabaaaniabggHiLdGcdaqadaqaamaalaaabaGaemizaqMaemivaq1aaSbaaSqaaiabd6gaUbqabaaakeaacqWGKbazcqWG0baDaaaacaGLOaGaayzkaaWaaWbaaSqabeaacqaIYaGmaaGccqGGSaalcaWLjaGaaCzcamaabmaabaGaeGymaeJaeGOmaidacaGLOaGaayzkaaaaaa@6B83@

where Ξ is a normalization constant and *β*_1_, *β*_2_, *ω *are Lagrange multipliers. The multipliers are determined by insuring that the constraints are satisfied. With this the most probable values of Λ, *T*, given the microarray data, are obtained by solving *∂ρ*/*∂*Λ = 0 coupled to *δρ*/*δ T*= 0, a functional differential equation for *T*(*t*) that we solve numerically (see appendix A). A discussion of a symmetry rule that applies to the invertibility of microarray data is given in appendix B and implies the need for a minimal amount of regulatory information in order to obtain a unique network. Figure 1 illustrates our approach where microarray and *a priori *TRN is used to infer TF activity time courses and TF/gene binding constants.

## Results and discussion

### Synthetic example

To test our implementation of the approach described above, and to find its practical limitations, we used a model network that consists of 20 genes and 10 TFs. None of the 20 genes is assumed to code for any of the 10 TFs. The TRN is shown in Table 1 where ± 1 implies up/down regulation. We took all binding constants and initial mRNA concentrations to be unity and 10^-9 ^*M*, respectively.

**Table 1:** The TRN used for the synthetic example.

	T1	T2	T3	T4	T5	T6	T7	T6	T9	T10
G1	-1	0	0	0	0	0	0	0	0	-1
G2	0	-1	0	0	0	0	0	0	0	1
G3	0	0	-1	0	0	0	1	0	0	0
G4	0	0	0	-1	0	0	0	0	0	0
G5	0	0	0	0	-1	0	0	0	0	0
G6	0	0	0	0	0	-1	0	0	0	0
G7	0	0	0	-1	0	0	-1	0	0	0
G8	0	0	0	0	0	0	0	-1	0	0
G9	0	0	0	0	0	0	0	0	-1	0
G10	0	0	0	0	0	-1	0	0	0	-1
G11	-1	0	0	0	0	0	0	0	0	-1
G12	0	-1	0	0	0	1	0	0	0	1
G13	0	0	-1	0	1	0	1	0	0	0
G14	0	0	0	-1	0	0	0	0	0	0
G15	-1	0	0	0	1	0	0	0	0	0
G16	0	0	0	0	0	1	0	0	0	0
G17	0	0	0	-1	0	0	-1	0	0	0
G18	0	0	0	0	0	0	0	1	0	0
G19	0	0	0	0	0	0	0	0	1	0
G20	0	0	0	0	0	1	0	0	0	1

Columns indicate the TFs whereas the rows indicate the genes that they affect (-1 for down regulation, +1 for up regulation, 0 for no interaction)

We generated TF time courses according to the following

*T*_*n*_(*t*) = 1 - 0.5sin(*υ*_*n*_*t *+ *ϕ*_*n*_),     (13)

where *υ*_*n*_, *ϕ*_*n *_are randomly chosen period and phases. Then we created the synthetic time series microarray data using our transcription model, selecting 10 "data points" that are 500 seconds apart. In the following, we demonstrate the robustness of our approach in reconstructing *T*(*t*) despite mistakes in the regulatory network and noise in the microarray data, conditions commonly encountered in practice.

### Uncertain regulatory network information

**Figure 2:**
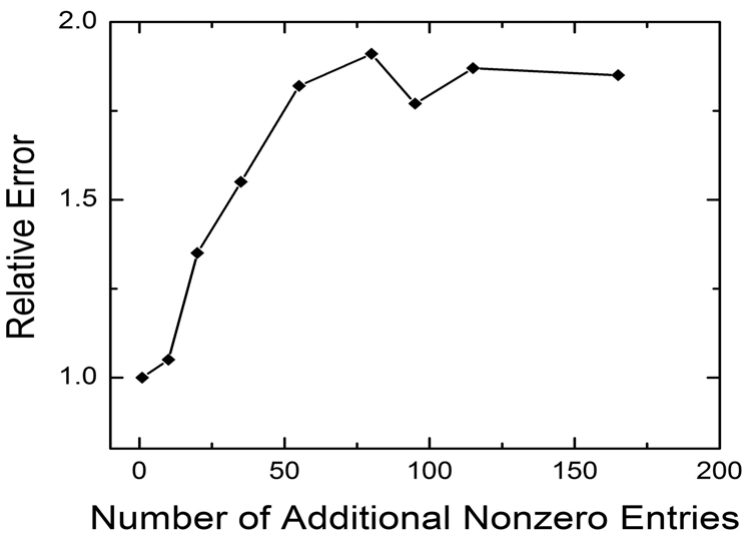
The sum of the square mismatch between the predicted and actual TF time course relative to the one obtained using the actual gene control network shown in Table 1. Although the mismatch increases as the number of interactions in the gene control network increases, it stays within a limit of 2 in this particular example, showing the potential of our approach to discover the operating gene control network hidden in a larger one (see Table 1 and 2 provided in the supplementary material).

Promoter sequence analysis can be used to determine the structure of the TRN based on likely binding sites. However, this approach is likely to suggest a large number of false positive interactions in the TRN. It is of interest to test whether our approach can filter the redundant nonzero entries in the control network. In our approach, if a TF is assumed to upregulate a gene, large binding constants, i.e. *QT *>> 1, imply that this interaction is unlikely (redundant) as *QT*/(1 + *QT*) ≈ 1. A similar argument hold for wrongly assumed down regulation as indicated by small binding constants (*QT *<< 1, and therefore 1/1 +*QT*) ≈ 1). Therefore, our methodology filters out incorrect interactions by assigning large/small binding constants for up/down regulation. To check the vulnerability of our approach to such redundant interactions, we added random nonzero factors in the regulatory network, and obtained the "conjectured full regulatory network" as shown in Table 2. As this network is full (i.e. each gene is regulated by all transcription factors), the NCA method fails. In our approach, the match between the predicted and know TF time courses is remarkable even when the "conjectured" full network was used. Figure 2 illustrates the effect of the number of redundant interactions on the mismatch between the predicted and actual TF time courses. The mismatch (relative to the one obtained using the actual network) does not exceed 2 even when the full interaction network is used as a starting point. Thus, our approach effectively filters the gene control network of unnecessary interactions.

**Table 2:** In order to test our approach for a large number of TF-gene interactions that might be suggested by sequence analysis or uncertain experimental data, we increased the number of interactions systematically by introducing additional random interactions.

	T1	T2	T3	T4	T5	T6	T7	T8	T9	T10
G1	-1	1	-1	1	1	1	1	-1	-1	-1
G2	1	-1	1	-1	-1	1	1	-1	1	1
G3	1	1	-1	-1	-1	-1	1	1	1	1
G4	1	1	-1	-1	1	-1	-1	1	-1	-1
G5	1	1	1	-1	-1	-1	-1	-1	1	-1
G6	-1	-1	1	1	-1	-1	1	1	-1	1
G7	1	-1	1	-1	1	-1	-1	-1	-1	1
G8	1	-1	1	1	-1	-1	-1	-1	1	1
G9	1	1	-1	-1	-1	1	1	-1	-1	1
G10	-1	1	1	1	-1	-1	1	1	-1	-1
G11	-1	-1	-1	1	-1	1	-1	1	-1	1
G12	-1	-1	1	1	1	1	-1	1	-1	1
G13	1	1	1	1	1	-1	1	-1	1	1
G14	1	-1	-1	-1	1	1	-1	1	-1	-1
G15	-1	1	1	-1	1	-1	*-*1	1	1	1
G16	-1	1	-1	1	1	1	1	-1	-1	-1
G17	1	1	1	-1	-1	1	1	1	-1	1
G18	1	1	1	-1	-1	1	1	1	-1	1
G19	1	-1	1	-1	-1	-1	*-*1	1	1	1
G20	-1	1	1	1	-1	1	*-*1	1	1	1

In the original matrix, there are 34 nonzero elements whereas in the augmented full matrix there are 200 nonzero elements. The challenge is to identify the actual operating TRN.

### Robustness to microarray error levels

Despite advances in the technology, microarray data has considerable levels of error. There have been few systematic analyses of microarray accuracy due to the many technology platforms available. These platforms have many technological variations which affect the accuracy and reproducibility of the measured expression levels of genes. Such variations are due to multiple techniques of making labeled material, various hybridization conditions, different microarray scanners and settings, etc [41]. Other factors that affect reproducibility of microarray experiments are variations in physiological conditions as well as the number of measurements made to improve the signal to noise ratio. Yeu *et al. *[42] estimated the coefficient of variation for non differentially expressed genes to be 12%–14%, and up to 25% for differentially expressed genes across the entire signal range using 10000 element *E. coli *cDNA microarray. Yuen *et al. *[43] reported a median coefficient of variation of 20.2% when they used cDNA triplicate measurements of 47 genes of *E. coli*. Novak et al. carried out an extensive study of *Affymetrix *Gene Chip oligonucleotide arrays using either identical RNA samples or RNA from replicate cultures under similar biological conditions [44]. They reported an overall coefficient of variation of 24.4% for 4377 genes of the IMR90 human cell line when they used 4 measurements on the same mRNA mixture sample. However, the overall coefficient of variation was 19.9% when they used 11 measurement of mRNA obtained from replicate cultures.

**Figure 3:**
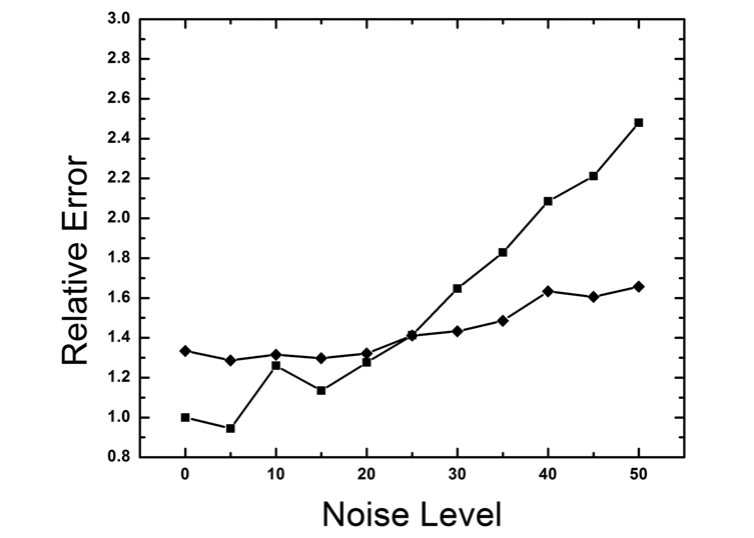
The sum of the square mismatch between the predicted and actual TF time course (relative to that obtained using noise-free microarray data) as a function of the amplitude of random noise added to the microarray data. The diamond and square markers represent the mismatch with and without the regularization constraint. For large noise levels the regularization constraint provides significant improvement in the calibration process.

In our implementation, we input raw microarray channel data, perform standard channel normalization (based on housekeeping genes determined using ranking of channel intensities and quality filtering for multiple spot and slide data [16]). The resulting confidence intervals constitute prior information about the level of noise in the microarray response. In this test, we investigate the vulnerability of our approach to error in microarray data. We added random noise to the synthetic microarray data that was obtained using the assumed TF time courses, and the transcription regulatory network (Table 1) as follows

miobs
 MathType@MTEF@5@5@+=feaafiart1ev1aaatCvAUfKttLearuWrP9MDH5MBPbIqV92AaeXatLxBI9gBaebbnrfifHhDYfgasaacH8akY=wiFfYdH8Gipec8Eeeu0xXdbba9frFj0=OqFfea0dXdd9vqai=hGuQ8kuc9pgc9s8qqaq=dirpe0xb9q8qiLsFr0=vr0=vr0dc8meaabaqaciaacaGaaeqabaqabeGadaaakeaacqWGTbqBdaqhaaWcbaGaemyAaKgabaGaem4Ba8MaemOyaiMaem4Camhaaaaa@33BA@ (*t*) = *m*_*i*_(*t*) × (1 + *noise *× (2*r *- 1)),     (14)

where *r *is a random number between 0 and 1. *noise *×100% represents the coefficient of variation or the percentage noise level in the microarray data. Figure 3 shows the mismatch (relative to the TF time course obtained without added noise) as a function of added noise level with and without the regularization constraint (see Eq. 10). The regularization constraint yields a better match at noise levels higher than 30%, providing robustness to the solution of the inverse problem when noisy data is used. More generally, the Lagrange multiplier for the regularization constraint, Eq. 12, should decrease with the width of the confidence interval. It should be noted that these results were obtained with a fixed Lagrange multiplier for the regularization constraint. If error level is believed to be very small, a smaller Lagrange multiplier should be used. We believe that error levels 30% and higher are to be expected in expression data, in particular for low concentration RNAs. This example also illustrates one of the advantages of time series over steady-state data in that the former is less vulnerable to noise due to the use of our regularization constraint.

### Healing mistakes in a proposed regulatory network

**Figure 4:**
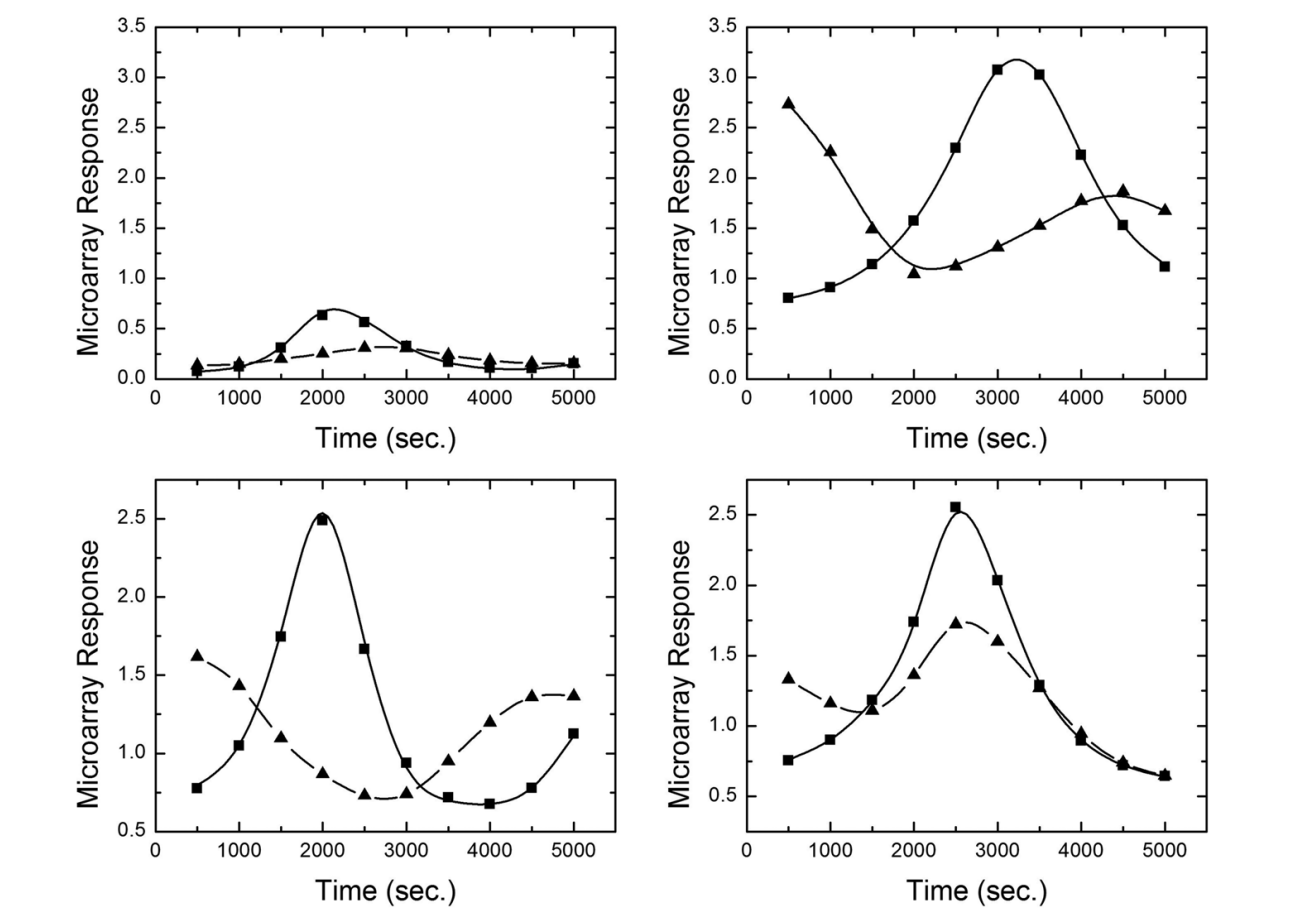
Comparison of observed (solid line) and predicted microarray data for genes 1–4 (see Table 1). Triangles indicate the best microarray fit when there are four mistakes in TRN (the first four entries in the upper left diagonal for genes 1–4); squares indicate the best microarray fit when we allow our program to correct the network. This shows that our algorithm is not only able to calculate the TF time courses, binding constants, etc.; it can also be used as a tool to decide whether a TF is up or down regulating a gene.

Often we rely on TF-gene interactions obtained from questionable quality resources. Therefore, it is important that our algorithm is robust to potential sign mistakes in the TRN due to regulatory differences between the cell line of interest and that for which the network was constructed. In this case, we run our code in a discovery mode that searches for network mistakes. We first rank genes based on the mismatch between the predicted and observed microarray response, highest ranked having the greatest mismatch. Then, we rank the TFs based on the rank and number of genes that they regulate. As calculation progresses, we periodically check the genes whose mismatch is greater than *E*_*average *_+ *aσ *where *E*_*average *_is the average mismatch and *σ *is the standard deviation of gene mismatch and *a *is an empirical parameter. Once the genes satisfying this criterion are identified, we change the sign of the regulatory interaction for each of the highest ranked TF (up/down). We also consider additional input that the user provides regarding confidence in each element of the TRN. At a given step in this process, we only change one sign per column of the TRN. After a few iterations, we monitor the mismatch behavior; if the sign change failed to improve the mismatch, we change the sign back. To test this algorithm, we took the TRN of Table 1 and introduced four mistakes by changing the sign of the diagonal elements of genes 1–4. Our algorithm successfully corrected the network after only 10 iterations. Figure 4 shows the predicted and observed microarray data. The triangle markers represent the best fit to the microarray data when the TRN with four mistakes is assumed, whereas the square markers represent the best fit after our algorithm corrected the mistakes. This demonstrates another aspect of our methodology, correcting a user-supplied TRN via microarray data. For large, real systems, we believe that many iterations will be necessary to arrive at an accurate network. The methodology will recommend changes in the TRN based on the microarray data and a user-suggested network. The rankings supplied with the improved network can be used to guide literature searches or carry out sequence analysis that can be used to further refine the network.

### Application to *E. coli*

**Figure 5:**
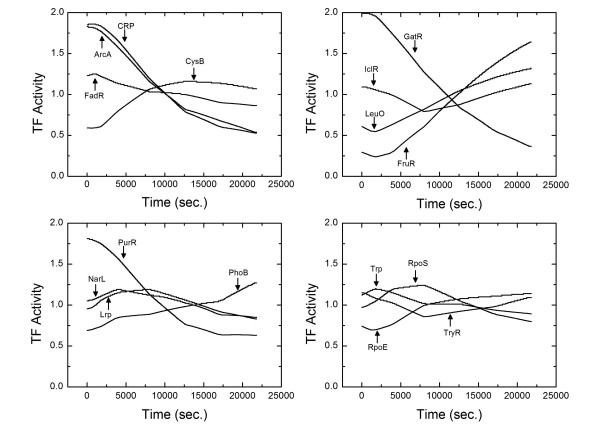
TF activity time courses for 16 of 38 TFs. These results are in qualitative agreement with those obtained by Kao et al. (2003) except for PhoB. pstC and pstS are upregulated by PhoB and their level of expression increases in time (shown in Figure 6), therefore one would expect the activity of PhoB to increase as well.

**Figure 6:**
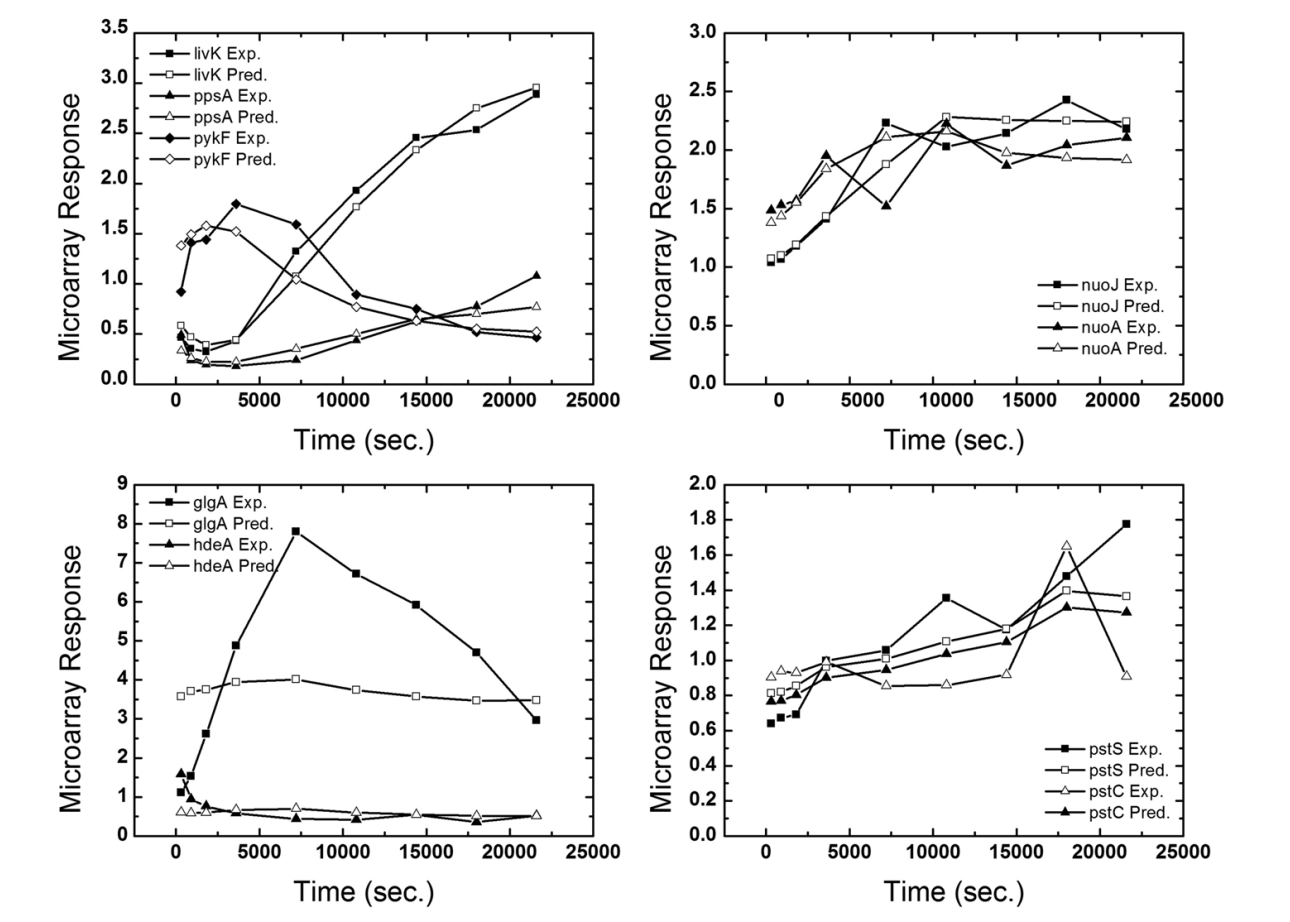
Comparison of predicted (hollow markers) and observed (solid markers) microarray response for a) nuoJ, nuoA, b) arcA, c) livK, ppsA, pykF, d) pstC and pstS.

To test our methodology we used the *E. coli *microarray data obtained for carbon source transition from glucose to acetate media. Details on the experimental conditions and the microarray procedure are provided in Ref. [29]. The data included expression levels (relative to initial time) of 100 genes at 300, 900, 1800, 3600, 7200, 10800, 14400, 18000 and 21600 seconds. The TRN used is based on *RegulonDB*[45] as modified by Kao et al. (2004). We made additional changes based on *EcoCyc *[24]. The final transcriptional regulatory network used is shown in Table 3. Figure 5 shows the time courses of 16 representative TF activities (out of 38). Kao et al. (2004) applied NCA to the same problem. However, the biologically relevant regulatory network that consists of 100 genes and 38 TFs does not satisfy the NCA column rank requirement. Furthermore, the transcription kinetics in our approach differs from the seady-state assumption and binding formulation used in NCA. Despite these differences, 15 out of 16 TF activity time courses (Kao et al. only presented the time courses of 16) are in qualitative agreement. As shown in Figure 5, PhoB increases as a result of response to acetate enrichment of the medium in contrast to the decreasing activity predicted by NCA. Therefore one would expect the phoB activity to increase as well. As a verification of our results, consider the arcA gene which makes ArcA TF that upregulates arcA itself. One would expect a correlation between the expression of arcA and TF activity of ArcA. To have an unbiased test, we took the expression of arcA from the microarray data and calculated the time course of ArcA activity based on the other 17 genes in the network that it regulates. The resultant ArcA time course was indistinguishable from the one obtained by including arcA in the microarray expression data. A comparison of predicted ArcA activity (Figure 5) and expression of arcA Figure 6 shows a similar trend. Figure 6 also shows a comparison between the predicted and observed microarray expression data for nuoJ, nuoA, arcA, livK, ppsA, pykF, pstC and pstS. In a second study, we added up to 30% noise to the *E. coli*. Microarray data, our approach is still found to be robust.

**Table 3:** The transcriptional regulatory network used in this study for the 100 responsive genes in E. coli when subjected to carbon source transition from glucose to acetate media.

**Gene**	**Transcription Factor**	**Gene**	**Transcription Factor**	**Gene**	**Transcription Factor**
AceA	-ArcA, -FruR, -IclR, +IHF	ldcC	+RpoS	trpA	-TrpR
AceB	-ArcA, -FruR, -IclR, +IHF	leuA	+LeuO	trpC	-TrpR
AceK	-ArcA, -FruR, -IclR, +IHF	leuB	+LeuO	tyrA	-TyrR
Acs	+Crp, +IclR, +RpoS, +FNR	leuC	+LeuO	tyrR	-TyrR
AdhE	-FruR, -NarL, +RpoS, +FIS	livJ	-Lrp,	ugpB	+Crp, +PhoB
aidB	+RpoS, +ada	livK	+FruR, -Lrp	ugpE	+Crp, +PhoB
arcA	+ArcA, +FNR	lrp	-Lrp, +RpoS, +GadE	uspA	-FadR
aroF	-TyrR	mdh	-ArcA, +Crp, -FlhD	wrbA	+RpoS
aroG	-TyrR	mdoH	+RpoE	xthA	+RpoS
aroM	-TrpR, -TyrR	mutH	+RpoS	yciG	+RpoS
aroP	-TyrR	narH	+NarL, +IHF, +FNR		
csgD	+CsgD, +OmpR	narl	+NarL, +IHF, +FNR		
csgE	+CsgD, +OmpR	narY	+RpoS		
csgF	+CsgD, +OmpR	nrfE	+NarL		
csiE	+Crp, +RpoS, +HNS	nuoA	-ArcA, +NarL, -IHF, -FNR		
cyoA	-ArcA, -FNR	nuoE	-ArcA, +NarL, -IHF, -FNR		
cyoB	-ArcA, -FNR	nuoF	-ArcA, +NarL, -IHF, -FNR		
cysA	+CysB	nuoH	-ArcA, +NarL, -IHF, -FNR		
cysH	+CysB	nuoJ	-ArcA, +NarL, -IHF, -FNR		
cysK	+CysB	osmE	-IHF		
cysM	+CysB	phoR	+PhoB, +TrpR		
dapA	+ RpoE	poxB	+RpoS, +MarA, +SoxS		
Epd	+Crp, +FruR	ppsA	+FruR		
fabA	+FadR	prop	+Crp, +RpoS, +FIS		
FtsZ	+RpoS, +SdiA, +RcsA	pspA	+IHF, +PspF, +RpoN		
gale	+Crp, -GalR, -Rob	pstC	+PhoB		
galK	+Crp, -GalR, -Rob	pstS	+PhoB		
galT	+Crp, -GalR, -Rob	purA	+RpoE		
gatA	-GatR	purK	-PurR		
gatC	-GatR	purM	-PurR		
gatD	-GatR	pykF	-FruR		
gatY	-GatR, +LeuO	rfaF	+RpoE		
glgA	+Crp	rob	+RpoS		
glgP	+Crp	rpoD	+RpoE, -Lexa		
glgS	+Crp	rpoE	+RpoE		
glpD	+Crp, -GlpR	rseC	+RpoE		
gltA	-ArcA, +Crp	sdhA	-ArcA, +Crp, -FNR, -FIS		
Gor	+OxyR	sdhB	-ArcA, +Crp, +NarL, -FNR, -FIS		
guaB	-PurR	serA	+Lrp		
hdeA	+RpoS, +GadX, +GadE	sucB	-ArcA, +Crp, -FNR, -FIS		
hdeB	+RpoS, +GadX, +GadE	sucC	-ArcA, +Crp, -FNR, -FIS		
IclR	-FadR	sucD	-ArcA, +Crp, -FNR, -FIS		
IlvB	+Crp	surA	+RpoE		
ilvH	+Lrp	tnaL	CAP		
Kbl	+Lrp	topA	+FIS		

+ indicates up-regulation

– indicates down-regulation

**Figure 7:**
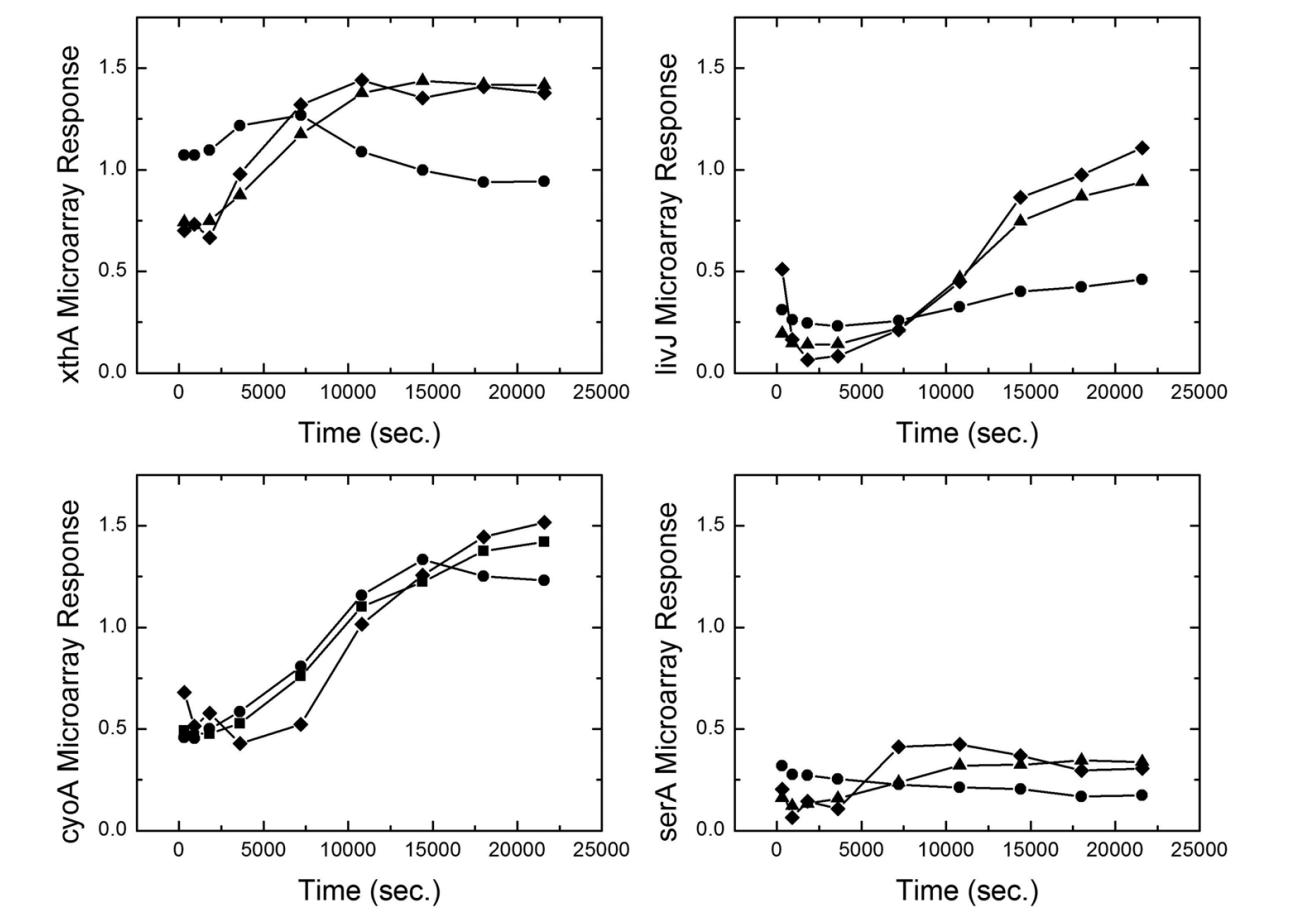
The predicted microarray response of xthA, livJ, cyoA and serA is enhanced after adding interactions suggested from promoter sequence analysis. Diamonds indicate the experimental microarray response; Circles indicate the predicted microarray response before adding the suggested interactions; Triangles indicate the predicted microarray response after adding the suggested interactions

**Figure 8:**
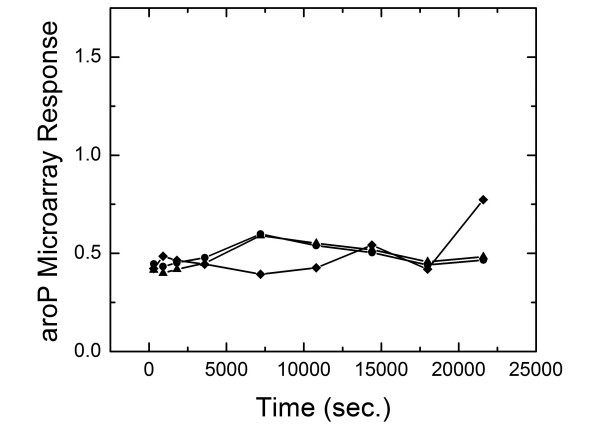
The predicted microarray response of aroP shows no significant improvement as we add the suggested CRP interaction. Diamonds indicate the experimental microarray response; Circles indicate the predicted microarray response before adding the suggested interactions; Triangles indicate the predicted microarray response after adding the suggested interactions.

Brown and Callan [33] have predicted many binding sites for the two TF CRP and ArcA. Among the genes included in our model, they predicted that two genes (xthA and livJ) in our data set to be regulated by ArcA. Also they predicted that two genes serA, cyoA and aroP are predicted to be regulated by CRP. To further examine the consistency of our approach with promoter sequence analysis, we performed another simulation after adding these interactions to the TRN obtained form *ecoCyc *and assuming the nature of these interactions (up vs down) to be unknown. Figure 7 shows improvements in the results obtained for xthA, livJ and serA. Our algorithm predicts that xthA and livJ are down regulated by ArcA. It also predicts that serA and cyoA are down regulated by CRP. However, no improvement is observed for aroP (Figure 8).

**Figure 9:**
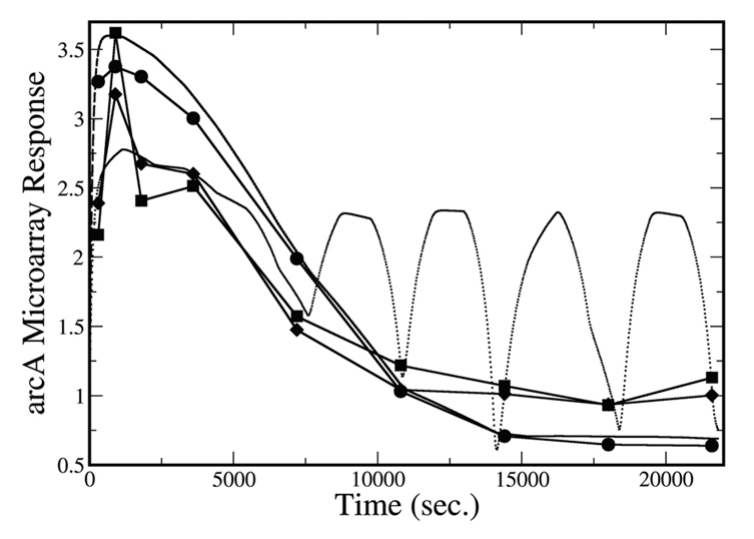
Comparison of predicted and observed microarray response for arcA. Diamonds indicate the experimental microarray response; squares indicate the experimental microarray response with 25% added noise; Circles indicate the predicted microarray response before adding 25% noise. Using data with 25% added noise, doted-line indicates the simulated microarray response when no regularization was imposed on the TF activity time courses, while the dashed-line is the microarray response when regularization was imposed on the TF activity time courses.

Regularization is important for discriminating between noise/data sparsity-related spurious oscillations and that arising from the nonlinear dynamics of transcription chemical kinetics [13,14]. To demonstrate the effect of regularization, we added 25% noise the observed microarray data. Figure 9, as an illustration, shows that arcA microarray response exhibit oscillatory behavior when no regularization constraint is used. These oscillations are not physical, but rather it is an artifact of using sparse noisy data.

### TRN discovery and limitations

A number of issues must be addressed in developing a TRN discovery strategy as follows. Discovering the structure and quantifying the physical chemistry of the gene regulatory network and the underlying mechanism has the challenges that arise in any chemical kinetics problem. For example, the simple process **A+B+C→ABC **can occur through the mechanism **A+B→AB**, **AB+C→ABC **or the other two permutations; to identify the actual mechanism, one must provide intermediate measurements on the dimers **AB**, **BC **and **CA**, rather than simply a measurement of the net rate of **ABC **production. Clearly, in a system with thousands of participating genes and hundreds of TFs, the resolution of the network and the detection of spurious ones, is a grand challenge of combinatorial magnitude. Essentially, all present network discovery methods suffer from this uniqueness difficulty due to the sparsity of available information. In this paper, we demonstrate how our method provides a way to augment an incomplete TRN and to identify inconsistencies in a proposed network based on microarray data.

Processes such as acetylation, methylation and phosphorylation, and the associated enzymes, play important roles in the wider set of pathways [34]. Thus the paradigm **genes→ mRNAs→ proteins → TFs → genes **.... is an oversimplification. While these processes could readily be added to our formulation, it is clear that data in addition to gene expression microarray observations would be required to resolve them. Thus we take the perspective that the simple paradigm cited above can be adopted as a starting point if it is recognized that other processes are somehow mediating the network we quantify. For example, if some genes are repressed in one mammalian cell line by methylation this will be reflected as a small transcriptional rate constant our approach reveals. When predicted levels for a given gene expression are found to be in poor agreement with observations and assuming that the probability that other TFs could be regulating that gene has already been explored, we consider this to imply that the simple paradigm has broken down and the other processes must be acting in a dynamical way to affect the gene expression time series. In light of the above, it is evident that network structure, physicochemical parameters and TF activity time courses can not all be extracted from a single approach.

## Conclusion

Multiplex time series data (e.g. microarray, ChIP-on-chip and protein mass spectroscopy) holds a great promise for the construction of the network of cellular processes and the calibration of the many associated physical chemical parameters. We have demonstrated these concepts in the context of transcription regulation understood through the analysis of microarray time series data. Casting the approach in a probabilistic framework has allowed us to address the uncertainties in microarray data. Our approach was found to be robust to error in the microarray data and mistakes in a proposed regulatory network. Our approach compliments other methods (e.g. gene ontology, phylogenetic and sequence analysis) when used as a part of a wider network discovery/quantification algorithm. Given its robustness, its capacity to refine and quantify complex networks of cellular processes, and the potential for extension to other multiplex bioanalytical data, we believe that our approach has great potential in the pure and applied life sciences.

## Availability and requirements

Project name: **KA**ryote **G**ene **AN**alyzer (KAGAN)

Project home page: 

Operating system(s): Windows (2000 and later versions) or Linux

Programming Languages: F77, php

Licence: a web interface that allows users to simulate their own data is available from the above site (free registration required).

Restrictions to non-academics: None

## Authors' contributions

AS, KT and PJO formulated the problem; and developed the theoretical model framework. AS and KT carried out the development, and implementation of the numerical algorithms. All authors participated in the writing of the manuscript, and have read and approved the manuscript.

## Appendices

### A. Numerical methods

The numerical methods used for simulating time evolution of mRNA populations, solving the calibration inverse problem by determining of TF time courses and model parameters are as follows. The latter parameters are sets of TF binding constants Q¯¯
 MathType@MTEF@5@5@+=feaafiart1ev1aaatCvAUfKttLearuWrP9MDH5MBPbIqV92AaeXatLxBI9gBaebbnrfifHhDYfgasaacH8akY=wiFfYdH8Gipec8Eeeu0xXdbba9frFj0=OqFfea0dXdd9vqai=hGuQ8kuc9pgc9s8qqaq=dirpe0xb9q8qiLsFr0=vr0=vr0dc8meaabaqaciaacaGaaeqabaqabeGadaaakeaacuWGrbqugaqhgaqhaaaa@2E1E@ and saturation limiting transcription rate coefficients *k*^*max *^and mRNA degradation rate constants *λ*.

Fast and accurate solution of the ODE model is crucial to construct thousands of gene expression levels to find the optimum model parameters in a practical time. For the *i*-th gene, mRNA population, *R*_*i*_, time evolution is computed using an implicit Euler method

Ri(tn+1)=Ri(tn)+Δtn+1ki(tn)1+λi.     (A.1)
 MathType@MTEF@5@5@+=feaafiart1ev1aaatCvAUfKttLearuWrP9MDH5MBPbIqV92AaeXatLxBI9gBaebbnrfifHhDYfgasaacH8akY=wiFfYdH8Gipec8Eeeu0xXdbba9frFj0=OqFfea0dXdd9vqai=hGuQ8kuc9pgc9s8qqaq=dirpe0xb9q8qiLsFr0=vr0=vr0dc8meaabaqaciaacaGaaeqabaqabeGadaaakeaacqWGsbGudaWgaaWcbaGaemyAaKgabeaakmaabmaabaGaemiDaq3aaSbaaSqaaiabd6gaUjabgUcaRiabigdaXaqabaaakiaawIcacaGLPaaacqGH9aqpdaWcaaqaaiabdkfasnaaBaaaleaacqWGPbqAaeqaaOWaaeWaaeaacqWG0baDdaWgaaWcbaGaemOBa4gabeaaaOGaayjkaiaawMcaaiabgUcaRiabfs5aejabdsha0naaBaaaleaacqWGUbGBcqGHRaWkcqaIXaqmaeqaaOGaem4AaS2aaSbaaSqaaiabdMgaPbqabaGcdaqadaqaaiabdsha0naaBaaaleaacqWGUbGBaeqaaaGccaGLOaGaayzkaaaabaGaeGymaeJaey4kaSccciGae83UdW2aaSbaaSqaaiabdMgaPbqabaaaaOGaeiOla4IaaCzcaiaaxMaadaqadaqaaiabbgeabjabc6caUiabigdaXaGaayjkaiaawMcaaaaa@5891@

The time step Δ*t*_*n*+1 _used is adaptive and depends on the maximum component of the rate vector *k*. The microarray expression level at a given experimental time is predicted as the relative abundance of mRNA populations to their reference state at initial time

miℓ=mi(tℓ)=Ri(tℓ)Ri(t0),ℓ=1,⋯Nmicro.(A.2)
 MathType@MTEF@5@5@+=feaafiart1ev1aaatCvAUfKttLearuWrP9MDH5MBPbIqV92AaeXatLxBI9gBaebbnrfifHhDYfgasaacH8akY=wiFfYdH8Gipec8Eeeu0xXdbba9frFj0=OqFfea0dXdd9vqai=hGuQ8kuc9pgc9s8qqaq=dirpe0xb9q8qiLsFr0=vr0=vr0dc8meaabaqaciaacaGaaeqabaqabeGadaaakeaafaqabeqacaaabaGaemyBa02aaSbaaSqaaiabdMgaPjabloriSbqabaGccqGH9aqpcqWGTbqBdaWgaaWcbaGaemyAaKgabeaakmaabmaabaGaemiDaq3aaSbaaSqaaiabloriSbqabaaakiaawIcacaGLPaaacqGH9aqpdaWcaaqaaiabdkfasnaaBaaaleaacqWGPbqAaeqaaOWaaeWaaeaacqWG0baDdaWgaaWcbaGaeS4eHWgabeaaaOGaayjkaiaawMcaaaqaaiabdkfasnaaBaaaleaacqWGPbqAaeqaaOWaaeWaaeaacqWG0baDdaWgaaWcbaGaeGimaadabeaaaOGaayjkaiaawMcaaaaacqGGSaalaeaacqWItecBcqGH9aqpcqaIXaqmcqGGSaalcqWIVlctcqWGobGtdaWgaaWcbaGaemyBa0MaemyAaKMaem4yamMaemOCaiNaem4Ba8gabeaaaaGccqGGUaGlcaWLjaWaaeWaaeaacqqGbbqqcqGGUaGlcqaIYaGmaiaawIcacaGLPaaaaaa@5D49@

In solving *∂ρ*/*∂*Λ = 0, a gradient steepest descent approach suffers from slow convergence. We overcome this via a combined steepest descent/simulated annealing approach. The key to efficiently solve the inverse problem cited above is to use an iterative alternating parameter approach. The calibration starts by minimization of the microarray error *E*^*cDNA *^with respect to TF binding constants Q¯¯
 MathType@MTEF@5@5@+=feaafiart1ev1aaatCvAUfKttLearuWrP9MDH5MBPbIqV92AaeXatLxBI9gBaebbnrfifHhDYfgasaacH8akY=wiFfYdH8Gipec8Eeeu0xXdbba9frFj0=OqFfea0dXdd9vqai=hGuQ8kuc9pgc9s8qqaq=dirpe0xb9q8qiLsFr0=vr0=vr0dc8meaabaqaciaacaGaaeqabaqabeGadaaakeaacuWGrbqugaqhgaqhaaaa@2E1E@. To reduce the computational cost, we utilize the exact solution of (Eq. 6) [46],

R0mi(t)exp⁡(λit)−R0=∫0tki(t′)exp⁡(λit′)dt′.     (A.3)
 MathType@MTEF@5@5@+=feaafiart1ev1aaatCvAUfKttLearuWrP9MDH5MBPbIqV92AaeXatLxBI9gBaebbnrfifHhDYfgasaacH8akY=wiFfYdH8Gipec8Eeeu0xXdbba9frFj0=OqFfea0dXdd9vqai=hGuQ8kuc9pgc9s8qqaq=dirpe0xb9q8qiLsFr0=vr0=vr0dc8meaabaqaciaacaGaaeqabaqabeGadaaakeaacqWGsbGudaWgaaWcbaGaeGimaadabeaakiabd2gaTnaaBaaaleaacqWGPbqAaeqaaOWaaeWaaeaacqWG0baDaiaawIcacaGLPaaacyGGLbqzcqGG4baEcqGGWbaCdaqadaqaaGGaciab=T7aSnaaBaaaleaacqWGPbqAaeqaaOGaemiDaqhacaGLOaGaayzkaaGaeyOeI0IaemOuai1aaSbaaSqaaiabicdaWaqabaGccqGH9aqpdaWdXaqaaiabdUgaRnaaBaaaleaacqWGPbqAaeqaaaqaaiabicdaWaqaaiabdsha0bqdcqGHRiI8aOWaaeWaaeaacuWG0baDgaqbaaGaayjkaiaawMcaaiGbcwgaLjabcIha4jabcchaWnaabmaabaGae83UdW2aaSbaaSqaaiabdMgaPbqabaGccuWG0baDgaqbaaGaayjkaiaawMcaaiabdsgaKjqbdsha0zaafaGaeiOla4IaaCzcaiaaxMaadaqadaqaaiabbgeabjabc6caUiabiodaZaGaayjkaiaawMcaaaaa@61F6@

The latter establishes an integral equation for *k*_*i*_(*t*). Solving for *k*_*i*_(*t*) at the given experimental microarray times yields a computationally efficient algebraic approach that allows the use of a simulated annealing algorithm [47] to find the optimum values for binding constants. The solution is achieved by discretizing the time profile of *k*_*i *_over a grid of microarray experimental times and then interpolating it as a continuous piecewise linear function,

ki(t)=kil+1−kiltl+1−tl(t−tl)+kil,tl<t<tl+1,l=0,⋯Nmicro.     (A.4)
 MathType@MTEF@5@5@+=feaafiart1ev1aaatCvAUfKttLearuWrP9MDH5MBPbIqV92AaeXatLxBI9gBaebbnrfifHhDYfgasaacH8akY=wiFfYdH8Gipec8Eeeu0xXdbba9frFj0=OqFfea0dXdd9vqai=hGuQ8kuc9pgc9s8qqaq=dirpe0xb9q8qiLsFr0=vr0=vr0dc8meaabaqaciaacaGaaeqabaqabeGadaaakeaafaqabeqadaaabaGaem4AaS2aaSbaaSqaaiabdMgaPbqabaGcdaqadaqaaiabdsha0bGaayjkaiaawMcaaiabg2da9maalaaabaGaem4AaS2aa0baaSqaaiabdMgaPbqaaiabdYgaSjabgUcaRiabigdaXaaakiabgkHiTiabdUgaRnaaDaaaleaacqWGPbqAaeaacqWGSbaBaaaakeaacqWG0baDdaWgaaWcbaGaemiBaWMaey4kaSIaeGymaedabeaakiabgkHiTiabdsha0naaBaaaleaacqWGSbaBaeqaaaaakmaabmaabaGaemiDaqNaeyOeI0IaemiDaq3aaSbaaSqaaiabdYgaSbqabaaakiaawIcacaGLPaaacqGHRaWkcqWGRbWAdaqhaaWcbaGaemyAaKgabaGaemiBaWgaaOGaeiilaWcabaGaemiDaq3aaSbaaSqaaiabdYgaSbqabaGccqGH8aapcqWG0baDcqGH8aapcqWG0baDdaWgaaWcbaGaemiBaWMaey4kaSIaeGymaedabeaakiabcYcaSaqaaiabdYgaSjabg2da9iabicdaWiabcYcaSiabl+Uimjabd6eaonaaBaaaleaacqWGTbqBcqWGPbqAcqWGJbWycqWGYbGCcqWGVbWBaeqaaaaakiabc6caUiaaxMaacaWLjaWaaeWaaeaacqqGbbqqcqGGUaGlcqaI0aanaiaawIcacaGLPaaaaaa@761E@

With (A.4) we can evaluate the above integral analytically

R0milobsexp⁡(λitl)−R0=∑s=0l−1(kis{exp⁡(λits+1)−exp⁡(λits)+λiexp⁡(λits)(ts+1−ts)λi2(ts+1−ts)}+kis+1{exp⁡(λits)−exp⁡(λits+1)+λiexp⁡(λits+1)(ts+1−ts)λi2(ts+1−ts)}).     (A.5)
 MathType@MTEF@5@5@+=feaafiart1ev1aaatCvAUfKttLearuWrP9MDH5MBPbIqV92AaeXatLxBI9gBaebbnrfifHhDYfgasaacH8akY=wiFfYdH8Gipec8Eeeu0xXdbba9frFj0=OqFfea0dXdd9vqai=hGuQ8kuc9pgc9s8qqaq=dirpe0xb9q8qiLsFr0=vr0=vr0dc8meaabaqaciaacaGaaeqabaqabeGadaaakeaacqWGsbGudaWgaaWcbaGaeGimaadabeaakiabd2gaTnaaDaaaleaacqWGPbqAcqWGSbaBaeaacqWGVbWBcqWGIbGycqWGZbWCaaGccyGGLbqzcqGG4baEcqGGWbaCdaqadaqaaGGaciab=T7aSnaaBaaaleaacqWGPbqAaeqaaOGaemiDaq3aaSbaaSqaaiabdYgaSbqabaaakiaawIcacaGLPaaacqGHsislcqWGsbGudaWgaaWcbaGaeGimaadabeaakiabg2da9maaqahabaWaaeWaaeaafaqaaeGabaaabaGaem4AaS2aa0baaSqaaiabdMgaPbqaaiabdohaZbaakmaacmqabaWaaSaaaeaacyGGLbqzcqGG4baEcqGGWbaCdaqadaqaaiab=T7aSnaaBaaaleaacqWGPbqAaeqaaOGaemiDaq3aaSbaaSqaaiabdohaZjabgUcaRiabigdaXaqabaaakiaawIcacaGLPaaacqGHsislcyGGLbqzcqGG4baEcqGGWbaCdaqadaqaaiab=T7aSnaaBaaaleaacqWGPbqAaeqaaOGaemiDaq3aaSbaaSqaaiabdohaZbqabaaakiaawIcacaGLPaaacqGHRaWkcqWF7oaBdaWgaaWcbaGaemyAaKgabeaakiGbcwgaLjabcIha4jabcchaWnaabmaabaGae83UdW2aaSbaaSqaaiabdMgaPbqabaGccqWG0baDdaWgaaWcbaGaem4CamhabeaaaOGaayjkaiaawMcaamaabmaabaGaemiDaq3aaSbaaSqaaiabdohaZjabgUcaRiabigdaXaqabaGccqGHsislcqWG0baDdaWgaaWcbaGaem4CamhabeaaaOGaayjkaiaawMcaaaqaaiab=T7aSnaaDaaaleaacqWGPbqAaeaacqaIYaGmaaGcdaqadaqaaiabdsha0naaBaaaleaacqWGZbWCcqGHRaWkcqaIXaqmaeqaaOGaeyOeI0IaemiDaq3aaSbaaSqaaiabdohaZbqabaaakiaawIcacaGLPaaaaaaacaGL7bGaayzFaaaabaGaey4kaSIaem4AaS2aa0baaSqaaiabdMgaPbqaaiabdohaZjabgUcaRiabigdaXaaakmaacmqabaWaaSaaaeaacyGGLbqzcqGG4baEcqGGWbaCdaqadaqaaiab=T7aSnaaBaaaleaacqWGPbqAaeqaaOGaemiDaq3aaSbaaSqaaiabdohaZbqabaaakiaawIcacaGLPaaacqGHsislcyGGLbqzcqGG4baEcqGGWbaCdaqadaqaaiab=T7aSnaaBaaaleaacqWGPbqAaeqaaOGaemiDaq3aaSbaaSqaaiabdohaZjabgUcaRiabigdaXaqabaaakiaawIcacaGLPaaacqGHRaWkcqWF7oaBdaWgaaWcbaGaemyAaKgabeaakiGbcwgaLjabcIha4jabcchaWnaabmaabaGae83UdW2aaSbaaSqaaiabdMgaPbqabaGccqWG0baDdaWgaaWcbaGaem4CamNaey4kaSIaeGymaedabeaaaOGaayjkaiaawMcaamaabmaabaGaemiDaq3aaSbaaSqaaiabdohaZjabgUcaRiabigdaXaqabaGccqGHsislcqWG0baDdaWgaaWcbaGaem4CamhabeaaaOGaayjkaiaawMcaaaqaaiab=T7aSnaaDaaaleaacqWGPbqAaeaacqaIYaGmaaGcdaqadaqaaiabdsha0naaBaaaleaacqWGZbWCcqGHRaWkcqaIXaqmaeqaaOGaeyOeI0IaemiDaq3aaSbaaSqaaiabdohaZbqabaaakiaawIcacaGLPaaaaaaacaGL7bGaayzFaaaaaaGaayjkaiaawMcaaaWcbaGaem4CamNaeyypa0JaeGimaadabaGaemiBaWMaeyOeI0IaeGymaedaniabggHiLdGccqGGUaGlcaWLjaGaaCzcamaabmaabaGaeeyqaeKaeiOla4IaeGynaudacaGLOaGaayzkaaaaaa@F317@

Our lack of knowledge about the initial value of *k*_*i *_(i.e.ki0
 MathType@MTEF@5@5@+=feaafiart1ev1aaatCvAUfKttLearuWrP9MDH5MBPbIqV92AaeXatLxBI9gBaebbnrfifHhDYfgasaacH8akY=wiFfYdH8Gipec8Eeeu0xXdbba9frFj0=OqFfea0dXdd9vqai=hGuQ8kuc9pgc9s8qqaq=dirpe0xb9q8qiLsFr0=vr0=vr0dc8meaabaqaciaacaGaaeqabaqabeGadaaakeaacqWGRbWAdaqhaaWcbaGaemyAaKgabaGaeGimaadaaaaa@3081@), gives us one less equation than the number of unknowns. The simplest way to overcome this difficulty is via a linear extrapolation between the points at *t*_2 _and *t*_1 _to the point at *t*_0_. Higher order extrapolations were tested and proven not to be very advantageous in this case, especially if we have frequent microarray measurements for early times. With this,

ki0=ki2−ki1t2−t1(t0−t1)+ki1.     (A.6)
 MathType@MTEF@5@5@+=feaafiart1ev1aaatCvAUfKttLearuWrP9MDH5MBPbIqV92AaeXatLxBI9gBaebbnrfifHhDYfgasaacH8akY=wiFfYdH8Gipec8Eeeu0xXdbba9frFj0=OqFfea0dXdd9vqai=hGuQ8kuc9pgc9s8qqaq=dirpe0xb9q8qiLsFr0=vr0=vr0dc8meaabaqaciaacaGaaeqabaqabeGadaaakeaacqWGRbWAdaqhaaWcbaGaemyAaKgabaGaeGimaadaaOGaeyypa0ZaaSaaaeaacqWGRbWAdaqhaaWcbaGaemyAaKgabaGaeGOmaidaaOGaeyOeI0Iaem4AaS2aa0baaSqaaiabdMgaPbqaaiabigdaXaaaaOqaaiabdsha0naaBaaaleaacqaIYaGmaeqaaOGaeyOeI0IaemiDaq3aaSbaaSqaaiabigdaXaqabaaaaOWaaeWaaeaacqWG0baDdaWgaaWcbaGaeGimaadabeaakiabgkHiTiabdsha0naaBaaaleaacqaIXaqmaeqaaaGccaGLOaGaayzkaaGaey4kaSIaem4AaS2aa0baaSqaaiabdMgaPbqaaiabigdaXaaakiabc6caUiaaxMaacaWLjaWaaeWaaeaacqqGbbqqcqGGUaGlcqaI2aGnaiaawIcacaGLPaaaaaa@536C@

(A.5) and (A.6) give us a linear system that can be solved for kil
 MathType@MTEF@5@5@+=feaafiart1ev1aaatCvAUfKttLearuWrP9MDH5MBPbIqV92AaeXatLxBI9gBaebbnrfifHhDYfgasaacH8akY=wiFfYdH8Gipec8Eeeu0xXdbba9frFj0=OqFfea0dXdd9vqai=hGuQ8kuc9pgc9s8qqaq=dirpe0xb9q8qiLsFr0=vr0=vr0dc8meaabaqaciaacaGaaeqabaqabeGadaaakeaacqWGRbWAdaqhaaWcbaGaemyAaKgabaGaemiBaWgaaaaa@30F4@, *l *= 0,⋯*N*_*micro*_. We use the resulting kil
 MathType@MTEF@5@5@+=feaafiart1ev1aaatCvAUfKttLearuWrP9MDH5MBPbIqV92AaeXatLxBI9gBaebbnrfifHhDYfgasaacH8akY=wiFfYdH8Gipec8Eeeu0xXdbba9frFj0=OqFfea0dXdd9vqai=hGuQ8kuc9pgc9s8qqaq=dirpe0xb9q8qiLsFr0=vr0=vr0dc8meaabaqaciaacaGaaeqabaqabeGadaaakeaacqWGRbWAdaqhaaWcbaGaemyAaKgabaGaemiBaWgaaaaa@30F4@ to construct a new error measure E˜
 MathType@MTEF@5@5@+=feaafiart1ev1aaatCvAUfKttLearuWrP9MDH5MBPbIqV92AaeXatLxBI9gBaebbnrfifHhDYfgasaacH8akY=wiFfYdH8Gipec8Eeeu0xXdbba9frFj0=OqFfea0dXdd9vqai=hGuQ8kuc9pgc9s8qqaq=dirpe0xb9q8qiLsFr0=vr0=vr0dc8meaabaqaciaacaGaaeqabaqabeGadaaakeaacuWGfbqrgaacaaaa@2DCE@,

E˜i=∑l=1Nmicro∑j=1Ns(i)(ln⁡((QijTnij(tl))1+bij21+QijTnij(tl))−ln⁡kil)2,     (A.7)
 MathType@MTEF@5@5@+=feaafiart1ev1aaatCvAUfKttLearuWrP9MDH5MBPbIqV92AaeXatLxBI9gBaebbnrfifHhDYfgasaacH8akY=wiFfYdH8Gipec8Eeeu0xXdbba9frFj0=OqFfea0dXdd9vqai=hGuQ8kuc9pgc9s8qqaq=dirpe0xb9q8qiLsFr0=vr0=vr0dc8meaabaqaciaacaGaaeqabaqabeGadaaakeaacuWGfbqrgaacamaaBaaaleaacqWGPbqAaeqaaOGaeyypa0ZaaabCaeaadaaeWbqaamaabmaabaGagiiBaWMaeiOBa42aaeWaaeaadaWcaaqaamaabmaabaGaemyuae1aaSbaaSqaaiabdMgaPjabdQgaQbqabaGccqWGubavdaWgaaWcbaGaemOBa42aaSbaaWqaaiabdMgaPjabdQgaQbqabaaaleqaaOWaaeWaaeaacqWG0baDdaWgaaWcbaGaemiBaWgabeaaaOGaayjkaiaawMcaaaGaayjkaiaawMcaamaaCaaaleqabaWaaSaaaeaacqaIXaqmcqGHRaWkcqWGIbGydaWgaaadbaGaemyAaKMaemOAaOgabeaaaSqaaiabikdaYaaaaaaakeaacqaIXaqmcqGHRaWkcqWGrbqudaWgaaWcbaGaemyAaKMaemOAaOgabeaakiabdsfaunaaBaaaleaacqWGUbGBdaWgaaadbaGaemyAaKMaemOAaOgabeaaaSqabaGcdaqadaqaaiabdsha0naaBaaaleaacqWGSbaBaeqaaaGccaGLOaGaayzkaaaaaaGaayjkaiaawMcaaiabgkHiTiGbcYgaSjabc6gaUjabdUgaRnaaDaaaleaacqWGPbqAaeaacqWGSbaBaaaakiaawIcacaGLPaaadaahaaWcbeqaaiabikdaYaaaaeaacqWGQbGAcqGH9aqpcqaIXaqmaeaacqWGobGtdaqhaaadbaGaem4CamhabaGaeiikaGIaemyAaKMaeiykaKcaaaqdcqGHris5aOGaeiilaWIaaCzcaiaaxMaadaqadaqaaiabbgeabjabc6caUiabiEda3aGaayjkaiaawMcaaaWcbaGaemiBaWMaeyypa0JaeGymaedabaGaemOta40aaSbaaWqaaiabd2gaTjabdMgaPjabdogaJjabdkhaYjabd+gaVbqabaaaniabggHiLdaaaa@86A5@

where *n*_*ij *_is the type of TF that binds to the *j*-th site on gene *i*. We find that, if the rate integral equation is accurately solved, then minimizing this error is equivalent to minimizing the microarray error. Applying simulated annealing enhance the likelihood that we get as close as needed to the global minimum of E˜
 MathType@MTEF@5@5@+=feaafiart1ev1aaatCvAUfKttLearuWrP9MDH5MBPbIqV92AaeXatLxBI9gBaebbnrfifHhDYfgasaacH8akY=wiFfYdH8Gipec8Eeeu0xXdbba9frFj0=OqFfea0dXdd9vqai=hGuQ8kuc9pgc9s8qqaq=dirpe0xb9q8qiLsFr0=vr0=vr0dc8meaabaqaciaacaGaaeqabaqabeGadaaakeaacuWGfbqrgaacaaaa@2DCE@_*i*_. When the resulting solution fails by increasing the error due to numerical instability, we switch to a steepest descent scheme.

For the TF activities we solve the discretized temporal regularization functional differential equations, *∂ρ*/*∂ T *= 0, with no flux boundary conditions [8] for its activity time course implicitly. For the *j*-th TF at the (*n*+1)-*th *iteration, one obtains

−ωΔsΔtf(Tjn+1(tl−1)+Tjn+1(tl+1))+(1+2ωΔsΔtf)Tjn+1(tl)=−Δs∂E∂Tjn(tl)+Tjn(tl),     (A.8)
 MathType@MTEF@5@5@+=feaafiart1ev1aaatCvAUfKttLearuWrP9MDH5MBPbIqV92AaeXatLxBI9gBaebbnrfifHhDYfgasaacH8akY=wiFfYdH8Gipec8Eeeu0xXdbba9frFj0=OqFfea0dXdd9vqai=hGuQ8kuc9pgc9s8qqaq=dirpe0xb9q8qiLsFr0=vr0=vr0dc8meaabaqaciaacaGaaeqabaqabeGadaaakeaadaWcaaqaaiabgkHiTGGaciab=L8a3jabfs5aejabdohaZbqaaiabfs5aejabdsha0naaBaaaleaacqWGMbGzaeqaaaaakmaabmaabaGaemivaq1aa0baaSqaaiabdQgaQbqaaiabd6gaUjabgUcaRiabigdaXaaakmaabmaabaGaemiDaq3aaSbaaSqaaiabdYgaSjabgkHiTiabigdaXaqabaaakiaawIcacaGLPaaacqGHRaWkcqWGubavdaqhaaWcbaGaemOAaOgabaGaemOBa4Maey4kaSIaeGymaedaaOWaaeWaaeaacqWG0baDdaWgaaWcbaGaemiBaWMaey4kaSIaeGymaedabeaaaOGaayjkaiaawMcaaaGaayjkaiaawMcaaiabgUcaRmaabmaabaGaeGymaeJaey4kaSYaaSaaaeaacqaIYaGmcqWFjpWDcqqHuoarcqWGZbWCaeaacqqHuoarcqWG0baDdaWgaaWcbaGaemOzaygabeaaaaaakiaawIcacaGLPaaacqWGubavdaqhaaWcbaGaemOAaOgabaGaemOBa4Maey4kaSIaeGymaedaaOWaaeWaaeaacqWG0baDdaWgaaWcbaGaemiBaWgabeaaaOGaayjkaiaawMcaaiabg2da9iabgkHiTiabfs5aejabdohaZnaalaaabaGaeyOaIyRaemyraueabaGaeyOaIyRaemivaq1aa0baaSqaaiabdQgaQbqaaiabd6gaUbaakmaabmaabaGaemiDaq3aaSbaaSqaaiabdYgaSbqabaaakiaawIcacaGLPaaaaaGaey4kaSIaemivaq1aa0baaSqaaiabdQgaQbqaaiabd6gaUbaakmaabmaabaGaemiDaq3aaSbaaSqaaiabdYgaSbqabaaakiaawIcacaGLPaaacqGGSaalcaWLjaGaaCzcamaabmaabaGaeeyqaeKaeiOla4IaeGioaGdacaGLOaGaayzkaaaaaa@8C5A@

(1+ωΔsΔtf)Tjn+1(t1)−ωΔsΔtfTjn+1(t2)=−Δs∂E∂Tjn(t1)+Tjn(t1),     (A.9)
 MathType@MTEF@5@5@+=feaafiart1ev1aaatCvAUfKttLearuWrP9MDH5MBPbIqV92AaeXatLxBI9gBaebbnrfifHhDYfgasaacH8akY=wiFfYdH8Gipec8Eeeu0xXdbba9frFj0=OqFfea0dXdd9vqai=hGuQ8kuc9pgc9s8qqaq=dirpe0xb9q8qiLsFr0=vr0=vr0dc8meaabaqaciaacaGaaeqabaqabeGadaaakeaadaqadaqaaiabigdaXiabgUcaRmaalaaabaacciGae8xYdCNaeuiLdqKaem4CamhabaGaeuiLdqKaemiDaq3aaSbaaSqaaiabdAgaMbqabaaaaaGccaGLOaGaayzkaaGaemivaq1aa0baaSqaaiabdQgaQbqaaiabd6gaUjabgUcaRiabigdaXaaakmaabmaabaGaemiDaq3aaSbaaSqaaiabigdaXaqabaaakiaawIcacaGLPaaacqGHsisldaWcaaqaaiab=L8a3jabfs5aejabdohaZbqaaiabfs5aejabdsha0naaBaaaleaacqWGMbGzaeqaaaaakiabdsfaunaaDaaaleaacqWGQbGAaeaacqWGUbGBcqGHRaWkcqaIXaqmaaGcdaqadaqaaiabdsha0naaBaaaleaacqaIYaGmaeqaaaGccaGLOaGaayzkaaGaeyypa0JaeyOeI0IaeuiLdqKaem4Cam3aaSaaaeaacqGHciITcqWGfbqraeaacqGHciITcqWGubavdaqhaaWcbaGaemOAaOgabaGaemOBa4gaaOWaaeWaaeaacqWG0baDdaWgaaWcbaGaeGymaedabeaaaOGaayjkaiaawMcaaaaacqGHRaWkcqWGubavdaqhaaWcbaGaemOAaOgabaGaemOBa4gaaOWaaeWaaeaacqWG0baDdaWgaaWcbaGaeGymaedabeaaaOGaayjkaiaawMcaaiabcYcaSiaaxMaacaWLjaWaaeWaaeaacqqGbbqqcqGGUaGlcqaI5aqoaiaawIcacaGLPaaaaaa@781F@

and

(1+ωΔsΔtf)Tjn+1(tNtimes)−ωΔsΔtfTjn+1(tNtimes−1)=−Δs∂E∂Tjn(tNtimes)+Tjn(tNTF).     (A.10)
 MathType@MTEF@5@5@+=feaafiart1ev1aaatCvAUfKttLearuWrP9MDH5MBPbIqV92AaeXatLxBI9gBaebbnrfifHhDYfgasaacH8akY=wiFfYdH8Gipec8Eeeu0xXdbba9frFj0=OqFfea0dXdd9vqai=hGuQ8kuc9pgc9s8qqaq=dirpe0xb9q8qiLsFr0=vr0=vr0dc8meaabaqaciaacaGaaeqabaqabeGadaaakeaadaqadaqaaiabigdaXiabgUcaRmaalaaabaacciGae8xYdCNaeuiLdqKaem4CamhabaGaeuiLdqKaemiDaq3aaSbaaSqaaiabdAgaMbqabaaaaaGccaGLOaGaayzkaaGaemivaq1aa0baaSqaaiabdQgaQbqaaiabd6gaUjabgUcaRiabigdaXaaakmaabmaabaGaemiDaq3aaSbaaSqaaiabd6eaonaaBaaameaacqWG0baDcqWGPbqAcqWGTbqBcqWGLbqzcqWGZbWCaeqaaaWcbeaaaOGaayjkaiaawMcaaiabgkHiTmaalaaabaGae8xYdCNaeuiLdqKaem4CamhabaGaeuiLdqKaemiDaq3aaSbaaSqaaiabdAgaMbqabaaaaOGaemivaq1aa0baaSqaaiabdQgaQbqaaiabd6gaUjabgUcaRiabigdaXaaakmaabmaabaGaemiDaq3aaSbaaSqaaiabd6eaonaaBaaameaacqWG0baDcqWGPbqAcqWGTbqBcqWGLbqzcqWGZbWCaeqaaSGaeyOeI0IaeGymaedabeaaaOGaayjkaiaawMcaaiabg2da9iabgkHiTiabfs5aejabdohaZnaalaaabaGaeyOaIyRaemyraueabaGaeyOaIyRaemivaq1aa0baaSqaaiabdQgaQbqaaiabd6gaUbaakmaabmaabaGaemiDaq3aaSbaaSqaaiabd6eaonaaBaaameaacqWG0baDcqWGPbqAcqWGTbqBcqWGLbqzcqWGZbWCaeqaaaWcbeaaaOGaayjkaiaawMcaaaaacqGHRaWkcqWGubavdaqhaaWcbaGaemOAaOgabaGaemOBa4gaaOWaaeWaaeaacqWG0baDdaWgaaWcbaGaemOta40aaSbaaWqaaiabdsfaujabdAeagbqabaaaleqaaaGccaGLOaGaayzkaaGaeiOla4IaaCzcaiaaxMaadaqadaqaaiabbgeabjabc6caUiabigdaXiabicdaWaGaayjkaiaawMcaaaaa@93A9@

Where *l *= 1,⋯(*N*_*times *_- 1), *ω *is the regularization coefficient and Δ*s *is chosen small enough by line search to assure that *E*^*cDNA *^is minimized. For the j-th set of equations, one must restrict the analysis to those genes regulated by that TF. The above linear system is efficiently solved using the Thomas algorithm for tridiagonal linear systems [48]. The remaining parameters (i.e. mRNA degradation rate coefficients *λ*, and transcription limiting rate *k*^*max*^) are found by a steepest descent based on *E*^*cDNA*^.

If only the microarray data was provided, and in absence of direct information and physical measurements on the binding constants and TF activities, it is clear that there is a degeneracy in the solution for this problem. This means that there are many states in the parameter space that have the similar *E*^*cDNA*^. For example if *Q*_*ij*_, Tnij
 MathType@MTEF@5@5@+=feaafiart1ev1aaatCvAUfKttLearuWrP9MDH5MBPbIqV92AaeXatLxBI9gBaebbnrfifHhDYfgasaacH8akY=wiFfYdH8Gipec8Eeeu0xXdbba9frFj0=OqFfea0dXdd9vqai=hGuQ8kuc9pgc9s8qqaq=dirpe0xb9q8qiLsFr0=vr0=vr0dc8meaabaqaciaacaGaaeqabaqabeGadaaakeaacqWGubavdaWgaaWcbaGaemOBa42aaSbaaWqaaiabdMgaPjabdQgaQbqabaaaleqaaaaa@325E@ satisfy the error minimization criterion, then for all *ε *> 0 also Q˜ij
 MathType@MTEF@5@5@+=feaafiart1ev1aaatCvAUfKttLearuWrP9MDH5MBPbIqV92AaeXatLxBI9gBaebbnrfifHhDYfgasaacH8akY=wiFfYdH8Gipec8Eeeu0xXdbba9frFj0=OqFfea0dXdd9vqai=hGuQ8kuc9pgc9s8qqaq=dirpe0xb9q8qiLsFr0=vr0=vr0dc8meaabaqaciaacaGaaeqabaqabeGadaaakeaacuWGrbqugaacamaaBaaaleaacqWGPbqAcqWGQbGAaeqaaaaa@30CA@ = *εQ*_*ij*_, T˜nij=1εTnij
 MathType@MTEF@5@5@+=feaafiart1ev1aaatCvAUfKttLearuWrP9MDH5MBPbIqV92AaeXatLxBI9gBaebbnrfifHhDYfgasaacH8akY=wiFfYdH8Gipec8Eeeu0xXdbba9frFj0=OqFfea0dXdd9vqai=hGuQ8kuc9pgc9s8qqaq=dirpe0xb9q8qiLsFr0=vr0=vr0dc8meaabaqaciaacaGaaeqabaqabeGadaaakeaacuWGubavgaacamaaBaaaleaacqWGUbGBdaWgaaadbaGaemyAaKMaemOAaOgabeaaaSqabaGccqGH9aqpdaWcaaqaaiabigdaXaqaaGGaciab=v7aLbaacqWGubavdaWgaaWcbaGaemOBa42aaSbaaWqaaiabdMgaPjabdQgaQbqabaaaleqaaaaa@3BDD@ satisfy the same criterion. A normalization procedure is imposed on the solution at every step in the iterative inversion by assuming knowledge of the temporal average of each TF to be T¯nij
 MathType@MTEF@5@5@+=feaafiart1ev1aaatCvAUfKttLearuWrP9MDH5MBPbIqV92AaeXatLxBI9gBaebbnrfifHhDYfgasaacH8akY=wiFfYdH8Gipec8Eeeu0xXdbba9frFj0=OqFfea0dXdd9vqai=hGuQ8kuc9pgc9s8qqaq=dirpe0xb9q8qiLsFr0=vr0=vr0dc8meaabaqaciaacaGaaeqabaqabeGadaaakeaacuWGubavgaqeamaaBaaaleaacqWGUbGBdaWgaaadbaGaemyAaKMaemOAaOgabeaaaSqabaaaaa@3276@ as follows

Tnijnorm(t)=Tnij(t)NmicroT¯nij∑l=1NmicroTnij(tl),     (A.11)
 MathType@MTEF@5@5@+=feaafiart1ev1aaatCvAUfKttLearuWrP9MDH5MBPbIqV92AaeXatLxBI9gBaebbnrfifHhDYfgasaacH8akY=wiFfYdH8Gipec8Eeeu0xXdbba9frFj0=OqFfea0dXdd9vqai=hGuQ8kuc9pgc9s8qqaq=dirpe0xb9q8qiLsFr0=vr0=vr0dc8meaabaqaciaacaGaaeqabaqabeGadaaakeaacqWGubavdaqhaaWcbaGaemOBa42aaSbaaWqaaiabdMgaPjabdQgaQbqabaaaleaacqWGUbGBcqWGVbWBcqWGYbGCcqWGTbqBaaGcdaqadaqaaiabdsha0bGaayjkaiaawMcaaiabg2da9maalaaabaGaemivaq1aaSbaaSqaaiabd6gaUnaaBaaameaacqWGPbqAcqWGQbGAaeqaaaWcbeaakmaabmaabaGaemiDaqhacaGLOaGaayzkaaGaemOta40aaSbaaSqaaiabd2gaTjabdMgaPjabdogaJjabdkhaYjabd+gaVbqabaGccuWGubavgaqeamaaBaaaleaacqWGUbGBdaWgaaadbaGaemyAaKMaemOAaOgabeaaaSqabaaakeaadaaeWbqaaiabdsfaunaaBaaaleaacqWGUbGBdaWgaaadbaGaemyAaKMaemOAaOgabeaaaSqabaGcdaqadaqaaiabdsha0naaBaaaleaacqWGSbaBaeqaaaGccaGLOaGaayzkaaaaleaacqWGSbaBcqGH9aqpcqaIXaqmaeaacqWGobGtdaWgaaadbaGaemyBa0MaemyAaKMaem4yamMaemOCaiNaem4Ba8gabeaaa0GaeyyeIuoaaaGccqGGSaalcaWLjaGaaCzcamaabmaabaGaeeyqaeKaeiOla4IaeGymaeJaeGymaedacaGLOaGaayzkaaaaaa@7278@

and

Qijnorm=Qij∑l=1NmicroTnij(tl)NmicroT¯nij.     (A.12)
 MathType@MTEF@5@5@+=feaafiart1ev1aaatCvAUfKttLearuWrP9MDH5MBPbIqV92AaeXatLxBI9gBaebbnrfifHhDYfgasaacH8akY=wiFfYdH8Gipec8Eeeu0xXdbba9frFj0=OqFfea0dXdd9vqai=hGuQ8kuc9pgc9s8qqaq=dirpe0xb9q8qiLsFr0=vr0=vr0dc8meaabaqaciaacaGaaeqabaqabeGadaaakeaacqWGrbqudaqhaaWcbaGaemyAaKMaemOAaOgabaGaemOBa4Maem4Ba8MaemOCaiNaemyBa0gaaOGaeyypa0ZaaSaaaeaacqWGrbqudaWgaaWcbaGaemyAaKMaemOAaOgabeaakmaaqahabaGaemivaq1aaSbaaSqaaiabd6gaUnaaBaaameaacqWGPbqAcqWGQbGAaeqaaaWcbeaakmaabmaabaGaemiDaq3aaSbaaSqaaiabdYgaSbqabaaakiaawIcacaGLPaaaaSqaaiabdYgaSjabg2da9iabigdaXaqaaiabd6eaonaaBaaameaacqWGTbqBcqWGPbqAcqWGJbWycqWGYbGCcqWGVbWBaeqaaaqdcqGHris5aaGcbaGaemOta40aaSbaaSqaaiabd2gaTjabdMgaPjabdogaJjabdkhaYjabd+gaVbqabaGccuWGubavgaqeamaaBaaaleaacqWGUbGBdaWgaaadbaGaemyAaKMaemOAaOgabeaaaSqabaaaaOGaeiOla4IaaCzcaiaaxMaadaqadaqaaiabbgeabjabc6caUiabigdaXiabikdaYaGaayjkaiaawMcaaaaa@6944@

This is self-consistent since it eliminates the cited above *QT *degeneracy.

### B. Symmetry rule and microarray inversion

For a general class of models used here, there is a TF up/down regulation symmetry that leads to a multiplicity in the determination of *T*(*t*). Notably there are 2NTF
 MathType@MTEF@5@5@+=feaafiart1ev1aaatCvAUfKttLearuWrP9MDH5MBPbIqV92AaeXatLxBI9gBaebbnrfifHhDYfgasaacH8akY=wiFfYdH8Gipec8Eeeu0xXdbba9frFj0=OqFfea0dXdd9vqai=hGuQ8kuc9pgc9s8qqaq=dirpe0xb9q8qiLsFr0=vr0=vr0dc8meaabaqaciaacaGaaeqabaqabeGadaaakeaacqaIYaGmdaahaaWcbeqaaiabd6eaonaaBaaameaacqWGubavcqWGgbGraeqaaaaaaaa@3163@ solutions of the microarray inversion problem that are equally viable unless some knowledge of b¯¯
 MathType@MTEF@5@5@+=feaafiart1ev1aaatCvAUfKttLearuWrP9MDH5MBPbIqV92AaeXatLxBI9gBaebbnrfifHhDYfgasaacH8akY=wiFfYdH8Gipec8Eeeu0xXdbba9frFj0=OqFfea0dXdd9vqai=hGuQ8kuc9pgc9s8qqaq=dirpe0xb9q8qiLsFr0=vr0=vr0dc8meaabaqaciaacaGaaeqabaqabeGadaaakeaacuWGIbGygaqhgaqhaaaa@2E40@ is provided. This is proved for our model as follows. The control function *H *(Eq. 2) contains factors of the form *x*^*b*^/(1 + *x*) where *b *is (*b*_*ij *_+ 1)/2 and *x *is *Q*_*ij *_Tnij
 MathType@MTEF@5@5@+=feaafiart1ev1aaatCvAUfKttLearuWrP9MDH5MBPbIqV92AaeXatLxBI9gBaebbnrfifHhDYfgasaacH8akY=wiFfYdH8Gipec8Eeeu0xXdbba9frFj0=OqFfea0dXdd9vqai=hGuQ8kuc9pgc9s8qqaq=dirpe0xb9q8qiLsFr0=vr0=vr0dc8meaabaqaciaacaGaaeqabaqabeGadaaakeaacqWGubavdaWgaaWcbaGaemOBa42aaSbaaWqaaiabdMgaPjabdQgaQbqabaaaleqaaaaa@325E@. Note that *x*/(1 + *x*) = 1/(1 + 1/*x*). Thus an up regulation with *Q*_*ij *_Tnij
 MathType@MTEF@5@5@+=feaafiart1ev1aaatCvAUfKttLearuWrP9MDH5MBPbIqV92AaeXatLxBI9gBaebbnrfifHhDYfgasaacH8akY=wiFfYdH8Gipec8Eeeu0xXdbba9frFj0=OqFfea0dXdd9vqai=hGuQ8kuc9pgc9s8qqaq=dirpe0xb9q8qiLsFr0=vr0=vr0dc8meaabaqaciaacaGaaeqabaqabeGadaaakeaacqWGubavdaWgaaWcbaGaemOBa42aaSbaaWqaaiabdMgaPjabdQgaQbqabaaaleqaaaaa@325E@ is equivalent to a down regulation with 1/(*Q*_*ij *_Tnij
 MathType@MTEF@5@5@+=feaafiart1ev1aaatCvAUfKttLearuWrP9MDH5MBPbIqV92AaeXatLxBI9gBaebbnrfifHhDYfgasaacH8akY=wiFfYdH8Gipec8Eeeu0xXdbba9frFj0=OqFfea0dXdd9vqai=hGuQ8kuc9pgc9s8qqaq=dirpe0xb9q8qiLsFr0=vr0=vr0dc8meaabaqaciaacaGaaeqabaqabeGadaaakeaacqWGubavdaWgaaWcbaGaemOBa42aaSbaaWqaaiabdMgaPjabdQgaQbqabaaaleqaaaaa@325E@). This suggests that unless for each TF we know *b*_*ij *_for at least one gene the inversion will allow two equally probable answers corresponding to *b*_*ij *_= ± 1 (either the correct result *Q*_*ij *_and Tnij
 MathType@MTEF@5@5@+=feaafiart1ev1aaatCvAUfKttLearuWrP9MDH5MBPbIqV92AaeXatLxBI9gBaebbnrfifHhDYfgasaacH8akY=wiFfYdH8Gipec8Eeeu0xXdbba9frFj0=OqFfea0dXdd9vqai=hGuQ8kuc9pgc9s8qqaq=dirpe0xb9q8qiLsFr0=vr0=vr0dc8meaabaqaciaacaGaaeqabaqabeGadaaakeaacqWGubavdaWgaaWcbaGaemOBa42aaSbaaWqaaiabdMgaPjabdQgaQbqabaaaleqaaaaa@325E@ for all *i *of the given TF type, or 1/Tnij
 MathType@MTEF@5@5@+=feaafiart1ev1aaatCvAUfKttLearuWrP9MDH5MBPbIqV92AaeXatLxBI9gBaebbnrfifHhDYfgasaacH8akY=wiFfYdH8Gipec8Eeeu0xXdbba9frFj0=OqFfea0dXdd9vqai=hGuQ8kuc9pgc9s8qqaq=dirpe0xb9q8qiLsFr0=vr0=vr0dc8meaabaqaciaacaGaaeqabaqabeGadaaakeaacqWGubavdaWgaaWcbaGaemOBa42aaSbaaWqaaiabdMgaPjabdQgaQbqabaaaleqaaaaa@325E@ with binding constant 1/*Q*_*ij*_). This implies that for each TF type *n *we must find at least one gene for which the nature of the regulation (up versus down) is known. This means that if b¯¯
 MathType@MTEF@5@5@+=feaafiart1ev1aaatCvAUfKttLearuWrP9MDH5MBPbIqV92AaeXatLxBI9gBaebbnrfifHhDYfgasaacH8akY=wiFfYdH8Gipec8Eeeu0xXdbba9frFj0=OqFfea0dXdd9vqai=hGuQ8kuc9pgc9s8qqaq=dirpe0xb9q8qiLsFr0=vr0=vr0dc8meaabaqaciaacaGaaeqabaqabeGadaaakeaacuWGIbGygaqhgaqhaaaa@2E40@ is written in a sparse form as a *N*_*g *_row by *N*_*TF *_column matrix, then at least one entry in each column must be known. (see Table 1).
